# Risk Factors for Violence in Psychosis: Systematic Review and Meta-Regression Analysis of 110 Studies

**DOI:** 10.1371/journal.pone.0055942

**Published:** 2013-02-13

**Authors:** Katrina Witt, Richard van Dorn, Seena Fazel

**Affiliations:** 1 Department of Psychiatry, University of Oxford, Warneford Hospital, Oxford, Oxfordshire, United Kingdom; 2 Research Triangle Institute International, Research Triangle Park, Durham, North Carolina, United States of America; Baylor College of Medicine, United States of America

## Abstract

**Background:**

Previous reviews on risk and protective factors for violence in psychosis have produced contrasting findings. There is therefore a need to clarify the direction and strength of association of risk and protective factors for violent outcomes in individuals with psychosis.

**Method:**

We conducted a systematic review and meta-analysis using 6 electronic databases (CINAHL, EBSCO, EMBASE, Global Health, PsycINFO, PUBMED) and Google Scholar. Studies were identified that reported factors associated with violence in adults diagnosed, using DSM or ICD criteria, with schizophrenia and other psychoses. We considered non-English language studies and dissertations. Risk and protective factors were meta-analysed if reported in three or more primary studies. Meta-regression examined sources of heterogeneity. A novel meta-epidemiological approach was used to group similar risk factors into one of 10 domains. Sub-group analyses were then used to investigate whether risk domains differed for studies reporting severe violence (rather than aggression or hostility) and studies based in inpatient (rather than outpatient) settings.

**Findings:**

There were 110 eligible studies reporting on 45,533 individuals, 8,439 (18.5%) of whom were violent. A total of 39,995 (87.8%) were diagnosed with schizophrenia, 209 (0.4%) were diagnosed with bipolar disorder, and 5,329 (11.8%) were diagnosed with other psychoses. Dynamic (or modifiable) risk factors included hostile behaviour, recent drug misuse, non-adherence with psychological therapies (*p* values<0.001), higher poor impulse control scores, recent substance misuse, recent alcohol misuse (*p* values<0.01), and non-adherence with medication (*p* value <0.05). We also examined a number of static factors, the strongest of which were criminal history factors. When restricting outcomes to severe violence, these associations did not change materially. In studies investigating inpatient violence, associations differed in strength but not direction.

**Conclusion:**

Certain dynamic risk factors are strongly associated with increased violence risk in individuals with psychosis and their role in risk assessment and management warrants further examination.

## Introduction

At least twenty studies have reported a positive association between schizophrenia and violence [1]. Less is known about risk and protective factors and the mechanisms mediating this relationship. Notable individual studies have also reported contrasting findings over the role of different factors such as threat/control override symptoms [2], [3], substance misuse [4], [5] and positive symptoms [6], [7].

Previous reviews also tend to emphasise different factors leading to a lack of consistency in developing clinically relevant violence assessment and management plans. For example, one narrative review emphasises the contribution of positive symptoms and comorbid substance misuse [8], whilst another emphasises the contribution of socio-demographic factors, theory of mind, and personality disorders in addition to positive symptoms and comorbid substance misuse [9]. Certain socio-demographic factors, however, were not associated with severe violence in a recent systematic review which found that a previous criminal history, a longer duration of untreated psychosis, and psychotic symptoms to be potentially important [10].

In addition, previous reviews have either focused on different questions or have not used systematic review methodology. Two have focused on whether psychosis increases the risk of violence compared to the general population [1], [11]. Of the reviews that have investigated risk factors for violence, two have been narrative reviews [9], [12] and one recent systematic review focussed on factors associated with violence in first-episode psychosis [10]. To date, however, no review has provided a synthesis of the relative importance of different risk and protective factors.

Clarification of the relative strength of risk and protective factors is important for 3 reasons: to assist in the development of evidence-based risk assessment approaches, to ensure treatment is targeted to the risk factors most likely to mitigate against violence risk whilst enhancing factors most protective against violence risk, and finally, to help understand the mechanisms that place certain individuals with psychosis at heightened violence risk compared to others. The area remains topical considering the increasing use of violence risk assessment instruments, which are used widely to inform decisions about legal responsibility and sentencing for individuals with mental illness [13], and to assist in determining the appropriateness and duration of detention of patients in secure psychiatric hospitals [14]. However, these approaches are resource-intensive and expensive, are not easily scalable particularly for low and middle income countries and, in the main, do not incorporate evidence from current research findings [15]. The lack of a cumulative and systematic evidence base to inform risk management in schizophrenia has been highlighted recently [16].

Therefore, we present a systematic review and meta-regression analysis of 110 studies to investigate the range of risk factors associated with violence in 45,553 individuals with schizophrenia or other psychoses. Additionally, as previous work has suggested that the risk factors for severe violence may differ from those for aggression or hostility [17], and between inpatients and outpatients [18], [19], two sub group analyses were conducted examining whether risk factors differed by severity of outcome and patient setting. Further, as we expect many individual factors to be highly correlated, we have synthesized similar factors using a meta-epidemiological approach that enabled us to provide information by broader domains such as psychopathological, positive symptoms, negative symptoms, treatment-related factors and others.

## Methods

Preferred Reporting Items for Systematic Reviews and Meta-Analyses (PRISMA) guidelines were followed [20].

### Search Strategy

We searched for studies indexed in six databases from their start dates: CINAHL (1 January 1982–31 December 2011), EBSCO (1 January 1980–31 December 2011), EMBASE (1 January 1980–31 December 2011), Global Health (1 January 1973–31 December 2011), PsycINFO (1 January1960–31 December 2011), PUBMED (1 January 1960–31 December 2011), as well as Google Scholar (1 January 2004–2011) using keywords that were inclusive for psychosis (e.g. schiz*, psych*, mental*) and violence (e.g. viol*, aggress*, crim*, offend*, danger*, hosti*). A full electronic search strategy for the CINAHL database is available in Figure S1. Reference lists were scanned by hand to identify additional studies. Non-English language articles were translated by post-graduate students for whom the relevant studies were in their first language. Corresponding authors were approached for clarification if there were uncertainties regarding either participant recruitment or methodology.

### Study Eligibility

Studies were included if: (a) diagnosis was made using either the Diagnostic and Statistical Manual (DSM) or International Classification of Diseases (ICD) criteria; (b) more than 95% of study participants were diagnosed with schizophrenia, schizophreniform disorder, schizoaffective disorder, delusional disorder, schizotypal disorder, psychosis not otherwise specified (but not transient psychoses such as drug-induced psychoses), and bipolar disorder [21]; (c) more than 95% of sample participants were aged eighteen years or older; (d) the study used a cohort, case-control, cross-sectional (including correlation and regression studies), or randomized-controlled trial (RCT) design; and, (e) the study investigated factors associated with a range of violent outcomes (aggression, hostility, or violent offending). The decision to use a 95% cut-off for criteria (b) and (c) was intended to make the findings specific to adults with psychosis rather than other psychiatric diagnoses and enabled the inclusion of 17 large-scale studies that would not have qualified for inclusion had a 100% cut-off been utilised. Once we took into account these criteria, our approach was inclusive in relation to study designs and samples order to gather the totality of evidence on this topic, and to use tests of heterogeneity to examine subgroups.

Given our emphasis on clinically relevant risk and protective factors, we have not reviewed studies of genetic and epigenetic associations with violence in psychosis. Studies were also excluded if they investigated only risk factors for childhood violence. Lastly, as the aim of this meta-analysis was to identify risk and protective factors for violence rather than for criminal offending, we excluded studies where samples did not differentiate between violent and non-violent offenders.

Through correspondence we were able to include new information from the following studies: Clinical Antipsychotic Trials of Intervention Effectiveness (CATIE) [22], Schizophrenia Care and Assessment Project (SCAP) [23], MacArthur Prevalence [24], 5 Site [25], the UK-700 [26], and one other recent report [27].

### Data Extraction

Analyses were conducted only on risk factors examined in three or more studies. This approach was adopted to improve validity of the risk estimates and to restrict the number of different risk factors reported (see Table S1 for an additional 77 factors with only two validations). Data was extracted by the first author using a standardised form. Where possible, risk and protective factors were separated into those that occurred within one year before assessment (“recent”) and those that occurred at any point (“history of”). This enabled separate ORs to be calculated for the proximal and distal variants of these factors. As proximal factors are more likely to be dynamic in nature, for example recent substance misuse is more likely to indicate on-going substance misuse than misuse occurring several years ago, this separation may enable the contribution of dynamic factors to be more clearly identified.

As some individual studies consisted of overlapping samples recruited over the same time frame, information on risk factors was preferentially extracted from the study with the largest sample size [28]. Data were only extracted from related studies when a new risk factor was reported.

The York criteria [29] was used to assess study quality. These consist of 10 items scoring the quality of cohort studies, and 9 for scoring case-control studies, which cover research design and data reporting. They include the following: random selection of cases and controls, comparability of cases and controls with regards to potential confounders, proportion of the sample successfully followed up, and comparability in the reasons for attrition between cases and controls. Specifically, studies were rated as poor quality if they satisfied three or fewer criteria, as moderate quality if they met between four and seven criteria, and high quality if eight or more criteria were met.

Data were converted to odds ratios (ORs) for the purposes of pooling using six approaches. If data were reported as frequencies or proportions, ORs were calculated directly. If data were reported continuously, log-transformed ORs were calculated from Cohen’s *d* [30]. If data were reported as correlation coefficients, these were converted to Cohen’s *d* [31] and then to log-transformed ORs. If data were reported as chi-square tests, these were converted to correlation coefficients [32], then to Cohen’s *d* and finally to log-transformed ORs. If data were reported as *z* scores, these were converted to correlation coefficients [31], then to Cohen’s *d* and finally to log-transformed ORs. Lastly, if data was reported as a Mann-Whitney *U* test, these were converted to a correlation coefficient following DeCoster (2009) [33], then to Cohen’s *d*, and finally to log-transformed ORs. Alongside ORs and accompanying 95% confidence intervals, for each risk factor the number of studies (*k*), the *z* score, the number of violent participants (*n* violent) and the total number of participants (*N* total) was also reported. Where ORs are reported in text, the following qualitative descriptions of the strength were used [34]: weak (OR 1.0–1.5), moderate (OR = 1.6–2.5), strong (OR = 2.6–9.9) and very strong (OR = 10.0 and above). As these categories are unlikely to apply to continuous variables where ORs of 1.0–1.5 may indicate at least moderate effects, we avoided using qualitative descriptions for the strength of the OR for factors measured continuously. All ORs were reported to one decimal place.

To assess for data extraction accuracy, comparison was made between two independent researchers (KW and KS) on a random sample of thirty-five studies. Concordance between these researchers regarding the proportion of the violent and non-violent groups exposed to a given risk or protective factor was very good (Cohen’s κ = 0.93) [35].

### Statistical Analyses

We used random effects models [36], which account for between-study heterogeneity by weighting studies similarly. Heterogeneity was assessed using the *I^2^* statistic, which represents the percentage of variance due to between-study factors rather than sampling error [37]. We used Peters’ [38] regression technique to examine publication bias as recommended for log transformed ORs, whilst Egger’s regression technique was used to examine publication bias for continuous variables [39]. All analyses were performed in STATA-IC, version 11.

### Meta-Epidemiological Domain Analyses

In addition to examining associations for individual risk and protective factors, similar factors were collapsed into one of ten psychosocial and clinical domains based on their classification in symptom checklists and potential modifiability. The ten domains were: demographic, premorbid, criminal history, psychopathological, positive symptomatology, negative symptomatology, neuropsychological, substance misuse, treatment-related, and suicidality. Random effects models were used to produce pooled estimates for each of these domains.

As some factors were indicative of increased risk, whilst others were indicative of reduced risk, factors were ranked on the basis of *z* scores rather than OR strength. To avoid double counting studies, where the same study provided more than one risk or protective factor per domain, data were included for the factor associated with the highest *z* score as this reflects the strength of the association as well as its precision. Studies using overlapping samples were excluded using the same approach outlined above.

### Analyses of Heterogeneity

Heterogeneity was examined by meta-regression when the *I^2^* statistic was greater than 75% [37]. Meta-regression explores whether a linear relationship exists between effect sizes and a given between-study characteristic [40]. Based on prior research [8], [9], [11], many between-study characteristics were examined, including: demographic and historical factors (age, gender, ethnicity, history of violence), diagnosis (schizophrenia vs. other psychoses; bipolar disorder vs. schizophrenia and other psychoses), study location (percentage of the sample treated as inpatients, outpatients, in prisons or in forensic psychiatric hospitals or units), study country (USA vs. rest of world), study quality, and violence source (register-based record of violence rather than self-report). When more than one characteristic was significantly (*p*<0.05) associated with heterogeneity, multivariate meta-regression was conducted to determine which study characteristics were independently associated with between-study heterogeneity.

In addition, we conducted two sensitivity analyses: (a) studies in which the outcome was severe violence rather than hostility or aggression (studies in which a psychometric measure of hostility or aggression was used to classify violent and non-violent participants), and (b) studies of inpatient violence. For the purposes of this review, a study was considered to be measuring inpatient violence when 95% or more of the sample were recruited from an inpatient setting.

## Results

### Study Characteristics

We included 110 studies of 73 independent samples that met review criteria. This involved 45,533 individuals, of whom 8,439 (18.5%) were violent (see Figure S2 for the search process, and Table S2 for methodological details and a reference list of included studies). A total of 39,995 (87.8%) were diagnosed with schizophrenia, 209 (0.4%) were diagnosed with bipolar disorder, and 5,329 (11.8%) were diagnosed with other psychoses. The average age of the participants was 35.8 years (*sd* = 5.6 years; range 21.1–54.3 years).

A total of 68 studies (61.8%) contained cases diagnosed with schizophrenia only; the remaining 42 (38.1%) included cases diagnosed with a range of psychotic illnesses (including schizophrenia). Of these 42 studies, 8 (19.0%) contained cases diagnosed with bipolar disorder. Violence was determined from register-based sources (e.g. Record of Arrest and Prosecution [RAP] sheets, criminal record, arrest or conviction registers) in 42 (38.1%) studies. Participants were recruited from a forensic psychiatric setting in 27 (24.5%) studies.

Mean sample size was 413.9 (*sd* = 1,404.7; range 16–13,806). The majority were case-control (*k = *70, *n* violent = 4,428, *N* total = 15,556). Studies were conducted in 27 countries: USA (*k = *29), UK (*k = *14), Israel (*k = *9), Australia (*k = *5), South Korea (*k = *5), Spain (*k = *5), Sweden (*k = *5), China (*k = *4), Germany (*k = *4), Mexico (*k = *4), Austria (*k = *3), Finland (*k = *2), Turkey (*k = *2) and one each from Brazil, Canada, Czech Republic, Denmark, Greece, India, Ireland, Japan, Norway, Singapore, South Africa, Taiwan, the Netherlands, and Tunisia. Five studies involved international collaborations. Previously unpublished tabular data from six studies (*n* violent = 756, *N* total = 3,646) were obtained specifically for this meta-analysis.

### Demographic and Premorbid Factors

A number of demographic factors were significantly associated with violence risk. Violence was strongly associated with a history of being violently victimized (OR = 6.1, 95% CI 4.0–9.1, *z = *8.7), moderately associated with recent homelessness (OR = 2.3, 95% CI 1.5–3.5, *z = *3.7), a history of homelessness (OR = 2.3, 95% CI 1.5–3.4, *z = *4.0), and being male (OR = 1.6, 95% CI 1.2–2.1, *z = *3.6), and weakly associated with non-white ethnicity (OR = 1.4, 95% CI 1.2–1.6, *z = *4.8), and a lower socio-economic status currently (OR = 1.4, 95% CI 1.1–1.9, *z = *3.0). (Table 1).

**Table 1:** Association between demographic factors and premorbid factors and risk of violence in individuals diagnosed with psychosis.

Risk Domain	Risk Factor	*k*	*n* Violent		*N* Total	Random Effects Pooled Odds Ratio *(95% CI)*		*Z*	*I^2^* (%)	Significance
**Demographic**										
History of violent victimization during adulthood		4	609	3,034		6.1	(4.0–9.1)	8.7	0	***
Recent homelessness		8	752	3,546		2.3	(1.5–3.5)	3.7	47	***
History of homelessness		9	910	4,254		2.3	(1.5–3.4)	4.0	40	***
Male		37	4,636	30,713		1.6	(1.2–2.1)	3.6	84	***
Non-white ethnicity		16	1,336	5,270		1.4	(1.2–1.6)	4.8	0	***
Lower socio-economic status currently		12	2,596	17,325		1.4	(1.1–1.9)	3.0	62	**
Received no more than a primary school education		3	138	649		1.4	(0.9–2.4)	1.5	0	
Lower family socio-economic status during childhood		3	209	778		1.4	(0.7–2.8)	1.0	50	
Lives in an urban environment currently		4	210	482		1.3	(0.9–1.9)	1.4	0	
Lives alone currently		9	602	2,907		1.2	(0.9–1.6)	1.5	18	
Received no more than a high school education		3	278	1,128		1.2	(0.6–2.5)	0.6	46	
Unmarried, widowed/divorced		25	3,121	20,773		1.1	(0.9–1.3)	1.6	18	
Unemployed currently		21	1,020	4,644		1.1	(0.8–1.6)	0.7	68	
Shorter duration of education (years)		16	845	3,194		1.1	(0.8–1.4)	1.0	0	
Lack any formal educational qualifications		6	366	2,416		1.1	(0.7–1.8)	0.7	54	
Younger age at study enrolment (years)		34	1,988	10,279		1.0	(0.9–1.1)	0.9	0	
Have children		3	1,965	14,775		1.0	(0.9–1.1)	0.9	0	
**Premorbid**										
Experienced physical abuse during childhood		4	444	2,177		2.2	(1.5–3.1)	4.4	39	***
Experienced sexual abuse during childhood		3	384	1,924		1.9	(1.5–2.4)	5.3	0	***
Parental history of criminal involvement		4	1,850	14,191		1.8	(1.5–2.2)	6.3	0	***
Parental history of alcohol misuse		5	1,871	14,209		1.6	(1.4–1.8)	6.7	0	***
Experienced the death of one parent during childhood		4	185	362		1.4	(0.7–2.6)	1.0	0	
Higher general premorbid adjustment scores		3	66	146		1.4	(0.6–3.3)	0.8	0	
Family history of mental illness (any type)		3	194	756		1.3	(0.8–2.1)	1.3	0	
History of head trauma		3	79	326		1.3	(0.6–2.5)	0.7	0	
Higher premorbid adjustment in childhood scores		3	66	146		1.2	(0.7–2.1)	0.8	0	
Higher premorbid adjustment in early adolescence scores		3	66	146		1.2	(0.7–1.9)	0.7	0	
Higher premorbid adjustment in late adolescence scores		3	66	146		1.0	(0.6–1.9)	0.2	4	
Experienced divorce/separation of parents during childhood, or raised by a single parent		4	185	362		0.7	(0.1–3.1)	0.4	79	

**Note:** *k* = number of studies analysed, *I^2^* = percentage of variability in effect size estimates that is attributable to between-study variation.

*** = significant to the 0.001 level.

** = significant to the 0.01 level.

* = significant to the 0.05 level. Factors ranked according to pooled OR magnitude.

In relation to premorbid factors, violence was moderately associated with reporting childhood physical (OR = 2.2, 95% CI 1.5–3.1, *z = *4.4) or sexual abuse (OR = 1.9, 95% CI 1.5–2.4, *z = *5.3), parental history of criminal involvement (OR = 1.8, 95% CI 1.5–2.2, *z = *6.3) and parental history of alcohol misuse (OR = 1.6, 95% CI 1.4–1.8, *z = *6.7). All other premorbid factors identified were not significant (Table 1).

### Criminal History Factors

Almost all criminal history factors were significantly associated with violence. Of those that were not, positive associations were found (Table 2).

**Table 2:** Association between criminal history factors and risk of violence in individuals diagnosed with psychosis.

Risk Factor	*k*	*n*Violent	*N*Total	Random EffectsPooled OddsRatio *(95% CI)*		*z*	*I^2^* (%)	Significance
History of assault	4	420	1,808	21.4	(5.2–86.6)	4.3	91	***
Higher scores on the Aggression Against Others subscale^1^	3	170	351	20.3	(0.5–770.1)	1.6	72	
Higher aggression scores	7	190	396	17.4	(2.6–117.0)	2.9	65	**
Higher psychopathy factor 2 scores	3	78	168	8.8	(1.6–46.7)	2.5	0	*
Higher psychopathy factor 1 scores	3	78	168	7.2	(1.4–35.9)	2.4	0	*
Higher scores on the Verbal Aggression subscale	5	181	456	5.5	(1.6–18.9)	2.7	12	**
History of imprisonment for any offence	6	644	2,990	4.5	(2.7–7.7)	5.6	62	***
Higher psychopathy total scores	7	183	486	4.4	(1.2–15.6)	2.3	58	*
Recent arrest for any offence	3	451	2,326	4.3	(2.7–6.7)	6.4	55	***
Aggressive behaviour during the study period	4	122	1,282	4.3	(1.2–15.1)	2.2	88	*
History of conviction for a violent offence	6	2,086	16,409	4.2	(2.2–9.1)	4.2	86	***
Meets criteria for psychopathy	4	69	358	3.6	(1.0–12.4)	2.0	8	*
History of conviction for any offence	5	194	856	3.5	(1.2–10.6)	2.2	67	*
History of arrest for any offence	4	510	2,781	3.5	(2.1–5.8)	4.9	72	***
History of violent behaviour	11	463	2,626	3.1	(2.2–4.4)	6.6	0	***
Greater number of previous arrests for any offence	3	73	268	3.0	(0.9–10.0)	1.8	0	
Hostility during the study period	3	318	2,724	2.8	(1.8–4.2)	4.9	0	***
Higher scores on the Aggression Against Objects subscale	4	170	436	1.9	(0.6–6.1)	1.1	44	
Recent violent behaviour	4	89	464	1.6	(0.8–3.0)	1.4	3	
Higher poor hostile and/or aggressive impulse control scores	3	114	259	1.5	(0.4–4.8)	0.6	29	
Higher hostility scores	16	701	3,290	1.5	(1.0–2.1)	2.2	1	*
History of conviction for a non-violent offence	4	477	5,137	1.4	(0.8–2.3)	1.2	30	
Younger age at first criminal offence (years)	3	247	1,047	1.2	(0.7–2.2)	0.8	0	

**Note:** *k* = number of studies analysed, *I^2^* = percentage of variability in effect size estimates that is attributable to between-study variation.

*** = significant to the 0.001 level.

** = significant to the 0.01 level.

* = significant to the 0.05 level. Factors ranked according to pooled OR magnitude.

1 When two small studies (Cheung, 1997c; Kim, 2009) were excluded, the association became: OR = 1.5, 95% CI 0.4–5.9, *z = *0.6, *I^2^ = *0%, *p* = 0.31, *k* = 1, *n* violent = 93, *N* Total = 186.

### Psychopathological, Positive and Negative Symptoms Factors

Violence was strongly associated with a lack of insight (OR = 2.7, 95% CI 1.4–5.2, *z = *2.9) and moderately associated with a diagnosis of comorbid antisocial personality disorder (OR = 2.1, 95% CI 1.0–4.3, *z = *2.0). Higher poor impulse control scores (OR = 3.3, 95% CI 1.5–7.2, *z = *3.1), higher general symptom scores (OR = 1.7, 95% CI 1.1–2.6, *z = *2.4), and higher total Positive And Negative Symptom Scale (PANSS) [41] scores (OR = 1.5, 95% CI 1.0–2.2, *z = *2.2) were also associated with violence.

With regards to positive symptoms, violence was associated with higher excitement scores (OR = 1.6, 95% CI 1.0–2.6, *z = *2.1), and higher positive symptoms scores (OR = 1.2, 95% CI 1.0–1.5, *z = *1.8). Violence was not significantly associated with any of the negative symptomatology factors identified (Table 3).

**Table 3:** Association between psychopathological, positive symptom and negative symptom factors and risk of violence in individuals diagnosed with psychosis.

Risk Domain	Risk Factor	*k*	*n*Violent	*N*Total	Random EffectsPooled OddsRatio *(95% CI)*				*z*	*I^2^* (%)	Significance	
**Psychopathological Symptoms**												
Higher poor impulse control scores		11	475	2,451	3.3	(1.5–7.2)			3.1	31	**	
Higher preoccupation scores		3	51	247	2.9	(0.9–9.5)			1.8	0		
Lacks insight (dichotomous)		6	280	2,402	2.7	(1.4–5.2)			2.9	61	**	
Higher scores on the Lack of Insight into Mental Disorder subscale		3	131	363	2.2	(0.8–6.3)			1.5	0		
Diagnosed with comorbid antisocial personality disorder		4	83	405	2.1	(1.0–4.3)			2.0	15	*	
Diagnosed with delusional disorder		3	68	201	2.0	(0.2–19.0)			0.6	44		
Higher general symptoms scores		21	1,052	4,233	1.7	(1.1–2.6)			2.4	13	*	
Higher cognitive functioning scores		5	261	528	1.7	(0.9–3.3)			1.6	0		
Higher total PANSS scores		15	771	3,226	1.5	(1.0–2.2)			2.2	10	*	
Diagnosed with undifferentiated schizophrenia subtype		8	349	694	1.5	(0.6–3.9)			0.9	61		
Higher total Clinical Global Impression (CGI) scores		3	331	1,532	1.5	(0.5–4.3)			0.8	44		
Higher lack of insight/judgement scores		6	441	1,985	1.4	(0.9–2.4)			1.5	0		
Higher guilt scores		4	137	354	1.4	(0.8–2.6)			1.2	0		
Higher somatic concerns scores		5	435	2,425	1.3	(0.8–2.1)			1.2	0		
Lower depression/anxiety scores		5	104	595	1.3	(0.7–2.3)			0.8	0		
Higher trait anxiety scores		11	516	2,795	1.2	(0.8–1.8)			1.0	0		
Diagnosed with bipolar disorder		3	176	487	1.2	(0.7–2.0)			0.8	0		
Higher uncooperativeness scores		9	658	3,113	1.2	(0.8–1.9)			1.1	18		
Higher confusion/disorientation scores		5	792	1,275	1.1	(0.8–1.6)			0.8	0		
Higher activation scores		4	254	699	1.1	(0.7–1.8)			0.5	0		
Higher total BPRS scores		6	260	1,309	1.1	(0.6–2.0)			0.5	4		
Diagnosed with paranoid schizophrenia subtype		11	505	1,611	1.1	(0.7–1.7)			0.4	59		
Younger age at psychosis onset (years)		15	600	1,598	1.0	(0.8–1.3)			0.6	0		
Diagnosed with catatonic schizophrenia subtype		4	210	436	1.0	(0.3–3.3)			0.08	0		
Higher social interest scores		3	1,051	2,382	1.0	(0.6–1.7)			0.2	0		
Higher total MINI scores		4	216	760	1.0	(0.8–1.2)			0.0	0		
Diagnosed with schizophrenia		20	1,382	5,522	0.9	(0.7–1.2)			0.4	48		
Diagnosed with disorganised schizophrenia subtype		6	298	587	0.9	(0.4–2.2)			0.1	2		
Diagnosed with schizoaffective disorder		8	483	1,363	0.8	(0.3–1.7)			0.5	73		
Diagnosed with Psychosis Not Otherwise Specified		3	67	214	0.4	(0.1–1.2)			1.5	0		
Diagnosed with residual schizophrenia subtype		5	237	485	0.3	(0.05–1.7)			1.3	83		
**Positive Symptoms**												
Experienced paranoid thoughts		3	130	503	2.0		(0.7–5.9)		1.3	79		
Higher conceptual disorganisation scores		3	70	220	1.7		(0.7–3.9)		1.2	0		
Higher excitement scores		9	490	1,685	1.6		(1.0–2.6)		2.1	0	*	
Higher delusions scores		4	417	1,972	1.6		(0.6–4.2)		0.9	11		
Experienced persecutory delusions		4	109	448	1.6		(0.7–3.6)		1.1	69		
Acutely symptomatic		3	158	945	1.5		(0.6–3.5)		1.0	74		
Higher positive symptom scores		28	1,108	5,342	1.2		(1.0–1.5)		1.8	0	*	
Higher hallucinations scores		6	492	2,490	1.2		(0.7–1.9)		0.9	0		
Experienced threat/control override delusions		5	584	1,849	1.2		(0.9–1.7)		1.5	7		
Higher grandiosity scores		5	435	2,425	1.2		(0.8–1.8)		1.0	0		
Experienced delusions of control		4	202	514	1.2		(0.7–2.0)		0.6	51		
Higher suspiciousness/persecution scores		8	512	2,610	1.1		(0.8–1.4)		0.6	0		
Higher thought disorder/disturbance scores		6	385	863	1.1		(0.8–1.7)		0.7	0		
Experienced delusions (any type)		3	90	372	1.1		(0.6–2.1)		0.4	0		
Experienced auditory hallucinations		3	443	1,582	1.1		(0.6–1.9)		0.4	74		
Higher paranoia scores		3	29	256	1.1		(0.2–5.5)		0.1	45		
Experienced command hallucinations		3	77	283	1.0		(0.5–2.0)		0.1	0		
Experienced grandiose delusions		4	114	352	0.8		(0.3–1.9)		0.5	40		
**Negative Symptoms**												
Higher poor attention span scores		6	483	2,104	1.4			(0.8–2.6)	1.2	0		
Lower total Quality of Life scores		3	452	2,038	1.2			(0.7–2.2)	0.8	0		
Diagnosed with comorbid depression		4	139	1,948	1.1			(0.7–1.7)	0.4	0		
Higher blunted affect scores		3	80	367	1.1			(0.6–2.0)	0.3	0		
Higher depression scores		13	1,449	3,629	1.0			(0.8–1.3)	0.3	0		
Higher negative symptom scores		27	1,157	4,538	1.0			(0.9–1.2)	0.5	0		
Higher social withdrawal scores		3	61	180	1.0			(0.6–1.8)	0.2	0		
Lower psychosocial functioning scores		3	769	1,065	1.0			(0.8–1.2)	0.1	0		

**Note:** *k* = number of studies analysed, *I^2^* = percentage of variability in effect size estimates that is attributable to between-study variation. *** = significant to the 0.001 level.

** = significant to the 0.01 level.

* = significant to the 0.05 level. Factors ranked according to pooled OR magnitude.

### Neuropsychological Factors

None of the neuropsychological factors investigated were significantly associated with violent outcomes (results not shown). These factors included: lower total scores on the full scale Wechsler Adult Intelligence Scale (WAIS) [42], [43], lower scores on the performance subscale of the WAIS, lower total scores on the National Adult Reading Test (NART) [44], lower scores on the picture completion item of the WAIS, lower scores on the verbal subscale of the WAIS, and higher perseverative errors on the Wisconsin Card Sorting Test [45].

### Substance Misuse Factors

Violence was very strongly associated with a history of polysubstance misuse (OR = 10.3, 95% CI 2.5–41.5, *z = *3.3), strongly associated with a diagnosis of comorbid substance use disorder (OR = 3.1, 95% CI 1.9–5.0, *z = *4.5), and recent substance misuse (OR = 2.9, 95% CI 1.3–6.3, *z = *2.6), and moderately associated with a history of alcohol misuse (OR = 2.3, 95% CI 1.7–3.3, *z = *5.1), a history of substance misuse (OR = 2.2, 95% CI 1.6–2.9, *z = *5.6), recent alcohol misuse (OR = 2.2, 95% CI 1.3–4.0, *z = *2.9), recent drug misuse (OR = 2.2 95% CI 1.6–3.1, *z = *5.1), and a history of drug misuse (OR = 2.1, 95% CI 1.3–3.5, *z = *3.9). It was unclear if there was an association between violence and a history of cannabis misuse (OR = 1.3, 95% CI 0.7–2.4, *z = *0.8) (Table 4).

**Table 4:** Association between substance misuse factors and risk of violence in individuals diagnosed with psychosis.

Risk Factor	*k*	*n*Violent	*N*Total	Random EffectsPooled OddsRatio *(95% CI)*		*z*	*I^2^* (%)	Significance
History of polysubstance misuse	3	144	338	10.3	(2.5–41.5)	3.3	0	**
Comorbid substance use disorder diagnosis	9	530	5,333	3.1	(1.9–5.0)	4.5	50	***
Recent substance (alcohol and/or drug) misuse	5	130	476	2.9	(1.3–6.3)	2.6	54	**
History of alcohol misuse	19	2,907	18,549	2.3	(1.7–3.3)	5.1	63	***
History of substance (alcohol and/or drug) misuse	16	1,067	5,365	2.2	(1.6–2.9)	5.6	46	***
Recent alcohol misuse	7	554	2,139	2.2	(1.3–4.0)	2.9	52	**
Recent drug misuse	7	695	3,604	2.2	(1.6–3.1)	5.1	38	***
History of drug misuse	14	2,809	18,561	2.1	(1.3–3.5)	2.9	93	**
History of cannabis misuse	4	95	315	1.3	(0.7–2.4)	0.8	23	

**Note:** *k* = number of studies analysed, *I^2^* = percentage of variability in effect size estimates that is attributable to between-study variation.

*** = significant to the 0.001 level.

** = significant to the 0.01 level.

* = significant to the 0.05 level. Factors ranked according to pooled OR magnitude.

### Treatment-Related Factors

Non-adherence with psychological therapies was strongly associated with violence risk (OR = 6.7, 95% CI 2.4–19.2, *z = *3.6), and moderately associated with non-adherence with medication (OR = 2.0 95% CI 1.0–3.7, *z* = 2.1) (Table 5).

**Table 5:** Association between treatment-related factors and risk of violence in individuals diagnosed with psychosis.

Risk Factor	*k*	*n*Violent	*N*Total	Random EffectsPooled OddsRatio *(95% CI)*		*z*	*I^2^* (%)	Significance
Non-adherent with psychological therapies	3	49	118	6.7	(2.4–19.2)	3.6	31	***
Non-adherent with medication	9	377	1,472	2.0	(1.0–3.7)	2.1	63	*
Not prescribed antipsychotic medication (any type)	7	216	579	1.7	(0.7–4.5)	1.2	58	
Shorter duration of current inpatient admission (months)	4	179	411	1.6	(0.1–17.8)	0.3	76	
Shorter duration of current outpatient treatment (months)	3	443	2,379	1.4	(0.7–2.6)	1.0	0	
Younger age at first psychiatric inpatient admission (years)	4	95	350	1.2	(0.7–1.8)	0.8	0	
Higher antipsychotic dosage (chlorpromazine equivalent units)	8	267	619	1.1	(0.8–1.7)	0.8	0	
Greater number of previous psychiatric admissions	10	325	1,286	1.1	(0.8–1.5)	0.7	0	
Longer duration of untreated illness (weeks)	3	116	380	1.0	(0.7–1.5)	0.2	0	
Shorter duration of illness (years)	19	1,240	4,621	1.0	(0.8–1.3)	0.5	0	
Shorter duration of antipsychotic treatment (months)	4	312	1,506	1.0	(0.7–1.4)	0.3	0	
Lower total extrapyramidal side effect scores	5	410	1,960	1.0	(0.5–2.2)	0.1	15	

**Note:** *k* = number of studies analysed, *I^2^* = percentage of variability in effect size estimates that is attributable to between-study variation.

*** = significant to the 0.001 level.

** = significant to the 0.01 level.

* = significant to the 0.05 level. Factors ranked according to pooled OR magnitude.

A number of treatment-related factors are likely confounded by indication. These include: being currently treated as an inpatient (OR = 5.2, 95% CI 1.8–15.3, *z = *3.0, *p* = 0.002), being referred for treatment from the criminal justice system (OR = 4.1, 95% CI 1.3–13.4, *z = *2.4, *p = *0.01), currently receiving treatment under involuntary/leveraged conditions (OR = 3.8, 95% CI 2.2–6.5, *z = *4.9, *p*<0.001), a history of receiving treatment under involuntary/leveraged conditions (OR = 3.6, 95% CI 2.1–6.1, *z = *4.8, *p*<0.001), being prescribed depot rather than oral antipsychotic medication formulations (OR = 2.2, 95% CI 1.1–4.1, *z = *2.4, *p = *0.01), and being prescribed typical/conventional rather than atypical antipsychotic medications (OR = 1.8, 95% CI 0.8–3.9, *z = *1.4, *p = *0.14). To prevent these factors from artificially inflating the strength of the treatment-related domain, they were excluded from the meta-epidemiological analysis.

### Suicidality Factors

Violence was moderately associated with a history of previous suicide attempts (OR = 1.6, 95% CI 1.1–2.3, *z = *2.4). However, violence was not significantly associated with any of the other suicidality risk factors investigated (Table 6).

**Table 6:** Association between suicidality factors and risk of violence in individuals diagnosed with psychosis.

Risk Factor	*k*	*n* Violent	*N* Total	Random EffectsPooled OddsRatio *(95% CI)*		*z*	*I^2^* (%)	Significance
History of experiencing suicidal ideations	4	347	1,803	1.7	(0.8–3.4)	1.6	49	
History of previous suicide attempts	12	1,075	4,037	1.6	(1.1–2.3)	2.4	42	*
Higher scores on the Aggression Against the Self subscale	3	170	351	1.4	(0.5–4.1)	0.6	35	
History of self-harm	3	254	807	1.0	(0.4–2.8)	0.1	68	

**Note:** *k* = number of studies analysed, *I^2^* = percentage of variability in effect size estimates that is attributable to between-study variation.

*** = significant to the 0.001 level.

** = significant to the 0.01 level.

* = significant to the 0.05 level. Factors ranked according to pooled OR magnitude.

### Heterogeneity Analysis

A small number of between-study characteristics were associated with higher or lower risk estimates (Table 7). Only 4 characteristics were associated with heterogeneity for more than one factor: the proportion of the sample with prior violence histories, the proportion of the sample detained in forensic psychiatric settings, the study being conducted in the USA (vs. rest of the world), and use of a register-based (rather than self-report) violence source.

**Table 7:** Univariate meta-regression results for factors with an *I^2^* value of 75 percent or greater.

Risk Factor	Characteristic	Univariate Meta-Regression					Multivariate Meta-Regression				
		*β*	*se*		*p*		*β*			*se*	*p*
**Male**											
Register-based (rather than self-reported) outcome		0.6		0.3		0.03					
% sample detained in a forensic facility (continuous)		0.3		0.01		0.04		0.07	0.02		0.02
% sample previously violent (continuous)		−0.01		0.007		0.04		−0.03	0.01		0.03
Study country (USA vs. rest of world)		−0.5		0.2		0.04					
Diagnosed according to DSM criteria		−0.6		0.2		0.008					
**History of assault**											
% sample previously violent (continuous)		0.1		0.02		0.03					
**History of conviction (violent offence)**											
% sample previously violent (continuous)		−0.05		0.01		0.03					
**Behaves Aggressively**											
Study country (USA vs. rest of world)		−1.9		0.4		0.04					
**Prescribed typical/conventional antipsychotic medication**											
Register-based (rather than self-reported) outcome		3.7		0.8		0.007					
% sample detained in a forensic facility (continuous)		0.05		0.01		0.03					
**History of drug misuse**											
Sample size (per 100)		0.008		0.003		0.03					

**Note:** Only factors with significant univariate or multivariate meta-regression results are included.

### Publication Bias Analysis

For risk and protective factors measured dichotomously, Peters’ test was significant for 3 factors: a history of imprisonment for any offence (*ß = *118.2, *se* = 24.0, *p = *0.008), a history of substance misuse (*ß = *52.7, *se* = 20.3, *p = *0.02), and currently receiving treatment under involuntary/leveraged conditions (*ß = *90.4, *se* = 29.0, *p = *0.01). Egger’s test of publication bias was significant for 34 factors (see Table S3).

### Meta-Epidemiological Risk Domain Analyses

**Figure 1:**
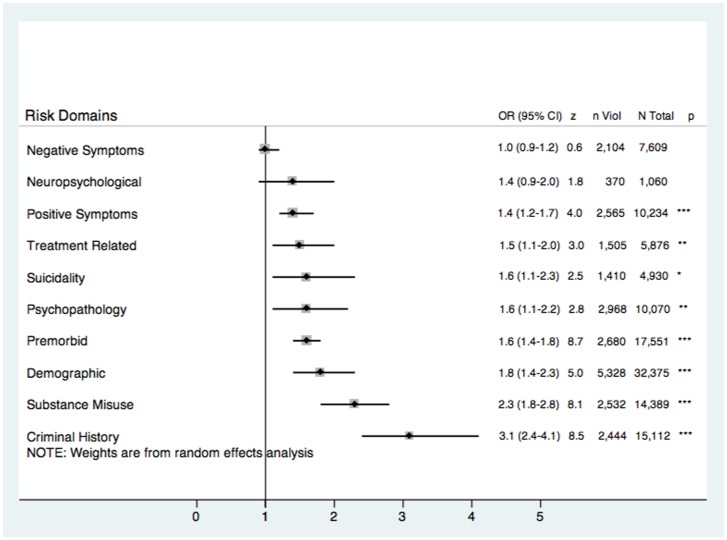
Risk of violence in psychosis reported as odds ratios (ORs) according to ten overall psychosocial and clinical domains (*k* = 110). *n* Violent = number of violent participants, *N* Total = total number of participants, *** = significant to the <0.001 level, ** = significant to the 0.01 level. * = significant to the 0.05 level. Factors ranked according to pooled OR magnitude.

Risk estimates were pooled to provide domain-specific random effect ORs (Figure 1). The strongest association by domain was for criminal history factors (OR = 3.1, 95% CI 2.4–4.1, *z = *8.5), followed by substance misuse (OR = 2.3, 95% CI 1.8–2.8, *z = *8.1), demographic (OR = 1.8, 95% CI 1.4–2.3, *z = *5.0), and premorbid (OR = 1.6, 95% CI 1.4–1.8, *z = *8.7) domains. Only two domains were not significantly associated with increased violence risk: negative symptoms and neuropsychological.

### Sensitivity Analyses

**Figure 2:**
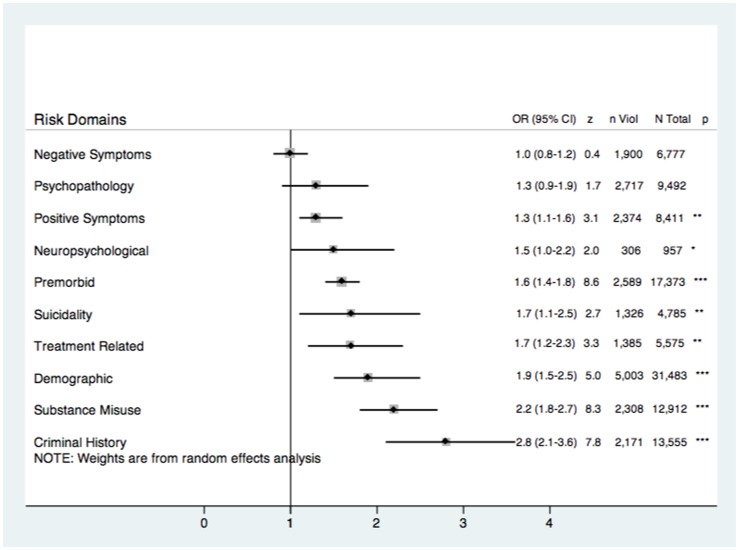
Risk of violence in psychosis reported as odds ratios (ORs) according to ten overall psychosocial and clinical domains for those studies which measured severe violence rather than aggression or hostility (*k* = 77). *n* Violent = number of violent participants, *N* Total = total number of participants, *** = significant to the <0.001 level, ** = significant to the 0.01 level. * = significant to the 0.05 level. Factors ranked according to pooled OR magnitude.

Seventy-seven (70.0%) studies measured severe violence rather than aggression or hostility. When domain-based analyses were restricted to these studies, the pattern and strength of associations mostly did not vary. The suicidality domain was more strongly associated with violence risk. The psychopathology domain was non-significantly associated with violence risk, whilst the neuropsychology domain was weakly associated with violence risk. The negative symptoms domain remained non-significantly associated with violence (Figure 2).

**Figure 3:**
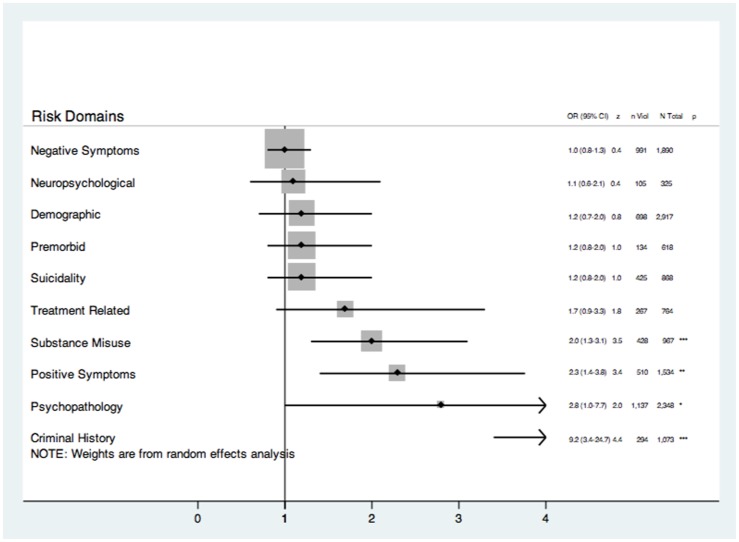
Risk of violence in psychosis reported as odds ratios (ORs) according to ten overall psychosocial and clinical domains for those studies conducted in predominately inpatient settings rather than predominately outpatient or mixed patient settings (*k* = 34). *n* Violent = number of violent participants, *N* Total = total number of participants, *** = significant to the <0.001 level, ** = significant to the 0.01 level. * = significant to the 0.05 level. Factors ranked according to pooled OR magnitude.

There were 34 (30.9%) studies based on inpatient samples and outcomes. When domain-based analyses were restricted to these studies, some differences emerged compared to the overall estimates. The substance misuse domain was less strongly associated with violence risk, although it remained significant. The psychopathology and positive symptoms domains were more strongly associated with violence risk. The negative symptoms, neuropsychological, demographic, premorbid, suicidality, and treatment-related domains were not significantly associated with violence risk (Figure 3).

## Discussion

This systematic review and meta-regression analysis of risk and protective factors for violent behaviour in adults with psychosis identified 110 studies of 73 independent samples and included a total of 45,533 individuals, 8,439 of whom were violent. We examined 146 individual risk and protective factors, and summarized them into ten domains.

The findings of this meta-analysis build on those of previous reviews in four major ways. First, this review found that a number of dynamic factors were significantly associated with violence risk including: hostile behaviour, poor impulse control, lack of insight, recent alcohol and/or drug misuse, and non-adherence with psychological therapies and medication. Despite a 2005 review suggesting the importance of dynamic factors for therapies aimed at risk reduction [46], the role of such factors has attracted relatively little primary research, and has rarely been emphasised in previous reviews [10].

Second, criminal history factors were more strongly associated with violence than substance misuse or demographic factors. Although this is an unsurprising finding, it provides some precision to the comparative importance of these factors. Moreover, it contrasts with recent reviews that have not differentiated the relative strength of these three domains [9], [10]. Our results are consistent, though, with several large epidemiological studies that suggest that criminal history factors such as previous violent behaviour and prior arrests are stronger predictors of risk [22], [25] compared with substance misuse [47] and certain demographic factors [48].

Third, we identified some potentially relevant treatment-related factors. In both the present analysis and a prior meta-analysis of nine studies in first-episode psychosis [10], receiving involuntary or leveraged treatment was associated with around a four-fold increase in violence risk. However, this is likely confounded by indication – that is, patients who receive certain treatments partly do so on the basis of violence risk [10], [24]._ENREF_19 This is also likely an explanation for our findings as to the association between violence and being prescribed depot antipsychotics as it has been suggested that patients at high risk of violence should be prescribed depot rather than oral formulations [49]. This finding highlights the need to use alternative study designs when comparing the effect of medication on violence risk.

Fourth, this review’s finding that negative symptoms were not significantly associated with violence risk is consistent with a previous review [10]. The lack of a statistical association between negative symptoms and violence risk suggests that motivation, and possibly planning, may mediate violence.

The role of atypical antipsychotics, which we found to be inversely but non-significantly associated with risk, also warrants further clarification. Although this finding contrasts with the CATIE trial [50], clozapine was not included in the first phase of this trial, whereas most studies contributing to this review did include clozapine as an atypical agent. As clozapine appears to have an anti-aggressive effect, a recent review has suggested that clozapine should be considered carefully for persistent aggression and violence [51]. In relation to the management of persistent violence, however, the contribution of other atypical antipsychotics remains uncertain [52]. Future research also is urgently needed to clarify whether antipsychotics reduce violence only in those individuals whose aggressive behaviour is driven by their psychotic symptomatology, or whether they lead to neurobiological changes that reduce violence independently of decreased psychotic symptoms [50]. Additionally, we found evidence that non-adherence with medication increased violence risk. Given the link between medication non-adherence and a range of adverse outcomes, this observation highlights the important role of treatment, and in particular therapies aimed at increasing treatment adherence [53].

Notably, among the strongest set of risk factors we identified in this review were related to victimization and, as others have reported, in individuals with schizophrenia the risk of being victimized is higher than the risk of violence perpetration [54]. Prior victimization may contribute to a “cycle of violence” whereby people with psychosis may be victimized because they place themselves in dangerous situations as a result of their own criminal behaviour [55].

We also found that previous suicide attempts were associated with violence risk. Although high levels of self-harm and suicide attempts are reported in individuals with psychosis [56], less research has focused on the relationship between suicide risk and violence in psychosis. Commonly used violence risk assessment instruments, however, do not routinely include assessment of suicide risk and may need revision in the light of this review. To our knowledge only one existing risk assessment instrument in adults, the Short Term Assessment of Risk and Treatability (START) [57], includes an assessment of suicidality. Suicidal ideation, however, was not significantly associated with violence risk. The reason that suicidal attempts rather than ideation are associated with violence suggests that impulsivity may be a mediator between suicidality and violence [58].

Risk factors did not change materially in strength and direction when focusing on severe violence. Whilst risk factors changed in strength when restricted to studies conducted in predominately inpatient settings, they did not change in direction. To our knowledge, these are novel findings and they would question the use of different risk assessment strategies for inpatients compared with community patients. In particular, the substance misuse domain remained moderately associated with violence regardless of patient setting. However, the positive symptoms and psychopathology domains did become more strongly associated with violence when analyses were limited to predominately inpatient samples.

Certain negative findings are notable. First, our review did not find an association between threat/control override (TCO) symptoms and violence risk. TCO symptoms refer to feeling threatened by others who are perceived as having violent intentions coupled with an override of self-control, possibly through the delusional perception of external forces. It may be that the inclusion of studies investigating aggression and hostility attenuated any association between TCO symptoms and violence in the present review.

Second, violence was not significantly associated with the neuropsychological domain. Previous work has suggested that neurological impairment contributes to aggression in individuals with psychosis [59]. In the current review, however, no neuropsychological factor appeared to be associated with increased violence risk. Given the degree of cognitive impairment already present in people with schizophrenia, the role played by neurobiological risk factors may be less important than originally thought. However, caution is warranted as there are theoretical reasons, and some case series data, to suggest that theory of mind deficits, lack of insight, and attitudinal cognitions may be associated with violence risk [60]. Furthermore, emerging neuroimaging studies support the role of frontal and inferior parietal activity in mediating violence risk in schizophrenia [61]. Further work, however, is necessary to determine the specificity of these findings.

### Strengths and Limitations

A strength of this review relates to the meta-epidemiological approach used to group similar factors into ten domains. This approach may enable clinicians and researchers to view violence risk more broadly in domains, rather than focusing on specific factors, many of which may overlap [48] and complicate the assessment of violence risk. A further strength was that we were able to include data from a number of large studies where the published reports did not provide enough information to be included in meta-analyses on risk factors. In part this was due to the use of effect size conversion formulae, which enabled data from studies that reported analyses continuously, rather than categorically, to be incorporated. Further, the use of such conversion formulae was not significantly associated with heterogeneity on meta-regression.

There are a number of important limitations to this review. Firstly, we grouped together diverse outcomes relating to violence. Although we felt this approach was necessary due to the lack of agreed outcome measures in the field [62], it may have contributed to the high levels of between-study heterogeneity observed for some risk factors. We attempted to address this limitation in a subgroup analysis that excluded studies in which the primary outcome measure was aggression and hostility rather than severe violence. Using this approach, no material difference in our overall findings was observed.

Further, we meta-analysed factors only when defined in a similar manner across three or more studies. Whilst this criterion may be more liberal than some may recommend, we felt it was necessary to adopt this approach to improve validity as there were so many individual factors reported in the primary studies. A similar approach has been used in large genetic association meta-analyses [63]. As a result of this criterion, however, some potentially relevant factors, for example those related to theory of mind, conduct disorder, and attention-deficit hyperactivity disorder were not included. Additionally, as we identified only two studies conducted in prison, our findings are not generalizable to custodial settings.

Another limitation is that our data should be interpreted in light of the fact that unadjusted ORs were reported as no consistent adjustments were made in the primary studies. Consequently, the present meta-analysis was unable to explore relationships between factors. Further work could provide more precise estimates by synthesizing individual participant data, which would allow for the calculation of adjusted effect sizes as well as the examination of the relationships between variables [64]. Finally, with the large number of factors identified, chance associations are to be expected.

Publication bias was significant for 37 of the 146 factors examined. However, the use of unpublished data was not significantly associated with between-study heterogeneity using meta-regression. This apparent contradiction may arise from the fact that many tests of publication bias are associated with inflated false-positive rates in meta-analyses of binary outcomes [65], but the possibility remains that some of the significant factors identified in this review were effected by publication bias. Larger, better quality studies should be conducted to clarify the factors for which there were uncertainty. Routine registration of observational studies will assist in the more careful investigation of sources of possible publication bias in future reviews [66]. As no method, to our knowledge, currently exists for assessing publication bias for both categorical and continuous outcomes, publication bias was not assessed for the meta-epidemiological domains.

Whilst meta-regression was used to explore which characteristics may be associated with between study heterogeneity, there were some characteristics we could not explore as the necessary data was not reported consistently in the primary studies, such as age at the violent index offence. Future research should include more background information on their samples. Lastly, as with all meta-analyses of observational data, causality cannot be determined.

### Implications

Whilst many of the premorbid and psychopathological risk and protective factors identified in this review – including being physically abused as a child and poor impulse control – are risk factors for violence in non-mentally ill populations [67], there are some differences specific to adults with psychotic disorders. For example, lower intelligence scores did not emerge as significantly associated with violence in this review despite its importance in the general population [67]. As discussed above, links between neuropsychological factors and violence may be attenuated in psychosis, where cognitive impairment is often already present.

In addition, the factors identified here are different from those for reoffending risk. Some demographic factors, such as young age and employment problems, are reported to be as important to the prediction of repeat offending as criminal history factors [48], a pattern that was not replicated in this review. In addition, we did not find that educational problems were associated with violence risk, unlike for repeat offending in mentally disordered offenders [48]. Further work is necessary to determine whether risk factors differ between offenders with psychosis and the general population, and the possible mechanisms involved.

The extent to which factors identified in this study are specific to violence in psychosis, rather than other mental disorders, remains unknown. Future research could compare risk and protective factors for violence between different psychiatric diagnoses, particularly in high risk groups such as those diagnosed with personality disorder [68] or substance misuse [1] as this will clarify whether unique risk assessment protocols are required for each diagnostic category.

Clarification of the direction and strength of risk and protective factors for violence in individuals with psychosis may enable researchers and clinicians to improve violence prediction and management, particularly in countries without specialist services or the resources to admit potentially dangerous patients for assessment. Alternative simpler approaches should be validated [69]. Further research also should investigate risk factors over longer follow-up as studies in this review were either cross-sectional or mostly followed patients for up to one or two years. The findings of new trials to improve adherence, especially through the use of financial incentives, may be important for violence risk reduction [70]. The role of antidepressant medication and anti-craving agents in the treatment of substance abuse comorbidity should also be considered [71].

Few studies have researched the impact of specific treatment interventions on violence risk, and we identified only three studies that investigated depot compared with oral medication. Where studies have investigated treatment effects, this has rarely been the primary focus of research. This is in contrast to research investigating the pharmacological treatment of suicide risk, where, for example, there have been over thirty trials of investigating lithium [72]. As many of the treatment interventions with the strongest associations with violence in this review are also potentially restrictive to individual liberty, research in this area will have implications for both policy and health service planning.

### Conclusions

This review confirms the strong association between criminal history and violence risk in psychosis and it also demonstrates that certain dynamic factors are potentially important for assessment and management of violence risk. These dynamic factors include: hostile behaviour, poor impulse control, lack of insight, general symptom scores, recent alcohol and/or drug misuse, and non-adherence with psychological therapies and medication. The potential contribution of addressing these factors would benefit from further examination in larger observational studies and large simple clinical trials [73] in which violence is a primary outcome.

## Supporting Information

Figure S1 **Full electronic search strategy for the CINAHL database depicting limits and Boolean key operators used as well as the number of “hits” for each search.**
(DOCX)

Figure S2 **Flow-chart depicting the search strategy employed to locate the 110 studies included in the systematic review and meta-analysis.**
(DOC)

Table S1 **Association between risk factors replicated in only two primary studies and risk of violence in individuals diagnosed with psychosis.**
(DOCX)

Table S2 **Methodological summary and reference list for the110 studies included in this systematic review and meta-analysis.**
(DOCX)

Table S3 **Beta coefficients, standard errors and probability values for the 34 risk and protective factors measured on a continuous scale in which Egger’s test of publication bias was significant.**
(DOCX)

## References

[1] Fazel S, Gulati G, Linsell L, Geddes JR, Grann M (2009) Schizophrenia and violence: Systematic review and meta-analysis. PLoS Med 6: 1–14. 10.1371/journal.pmed.1000120 PMC2718581 19668362

[2] Link BG, Stueve A, Phelan J (1998) Psychotic symptoms and violent behaviours: Probing the components of “threat/control-override” symptoms. Soc Psychiatry Psychiatr Epidemiol 33: s55–s60. 9857780 10.1007/s001270050210

[3] Appelbaum PS, Robbins PC, Monahan J (2000) Violence and delusions: Data from the MacArthur Violence Risk Assessment Study. Am J Psychiatry 157: 566–572. 10739415 10.1176/appi.ajp.157.4.566

[4] Van Dorn RA, Volavka J, Johnson N (2011) Mental disorder and violence: Is there a relationship beyond substance use? Soc Psychiatry Psychiatr Epidemiol 47: 487–503. 21359532 10.1007/s00127-011-0356-x

[5] Elbogen EB, Johnson SC (2009) The intricate link between violence and mental disorder: Results from the National Epidemiologic Survey on Alcohol and Related Conditions. Arch Gen Psychiatry 66: 152–161. 19188537 10.1001/archgenpsychiatry.2008.537

[6] Taylor PJ, Gunn J (1984) Violence and psychosis I: Risk of violence among psychotic men. BMJ 288: 1945–1949. 6428616 10.1136/bmj.288.6435.1945 PMC1442214

[7] Verma S, Poon LY, Subramaniam M, Chong S-A (2005) Aggression in Asian patients with first-episode psychosis. Int J Soc Psychiatry 51: 365–371. 16400911 10.1177/0020764005060852

[8] Walsh E, Buchanan A, Fahy T (2002) Violence and schizophrenia: Examining the evidence. Br J Psychiatry 180: 490–495. 12042226 10.1192/bjp.180.6.490

[9] Bo S, Abu-Akel A, Kongerslev M, Haahr UH, Simonsen E (2011) Risk factors for violence among patients with schizophrenia. Clin Psychol Rev 31: 711–726. 21497585 10.1016/j.cpr.2011.03.002

[10] Large M, Nielssen O (2011) Violence in first-episode psychosis: A systematic review and meta-analysis. Schizophr Res 125: 209–220. 21208783 10.1016/j.schres.2010.11.026

[11] Douglas KS, Guy LS, Hart SD (2009) Psychosis as a risk factor for violence to others: A meta-analysis. Psychol Bull 135: 679–706. 19702378 10.1037/a0016311

[12] Volavka J, Citrome LL (2011) Pathways to aggression in schizophrenia affect results of treatment. Schizophr Bull 37: 921–929. 21562140 10.1093/schbul/sbr041 PMC3160235

[13] Mercado CC, Ogloff JRP (2007) Risk and preventive detention of sex offenders in Australia and the United States. Int J Law Psychiatry 30: 49–59. 17157911 10.1016/j.ijlp.2006.02.001

[14] Khiroya R, Weaver T, Maden T (2009) Use and perceived utility of structured violence risk assessments in English medium secure forensic units. Psychiatrist 33: 129–132.

[15] Singh JP, Fazel S (2010) Forensic risk assessment: A metareview. Crim Justice Behav 27: 965–986.

[16] Wehring HJ, Carpenter WT (2011) Violence and schizophrenia. Schizophr Bull 37: 877–878. 21860032 10.1093/schbul/sbr094 PMC3160236

[17] Raja M, Azzoni A (2005) Hostility and violence of acute psychiatric inpatients. Clinical Practice and Epidemiology in Mental Health 1: 11–19. 16053528 10.1186/1745-0179-1-11 PMC1188062

[18] Steinert T (2002) Prediction of inpatient violence. Acta Psychiatr Scand 106: 133–141. 10.1034/j.1600-0447.106.s412.29.x 12072145

[19] Arango C, Barba AC, González-Salvador T, Ordóñez AC (1999) Violence in inpatients with schizophrenia: A prospective study Schizophr Bull. 25: 493–503. 10.1093/oxfordjournals.schbul.a033396 10478784

[20] Liberati A, Altman DG, Tetzlaff J, Mulrow C, Gøtzsche PC, et al (2009) The PRISMA statement for reporting systematic reviews and meta-analyses of studies that evaluate health care interventions: Explanation and elaboration. PLoS Med 6: e1000100. 19621070 10.1371/journal.pmed.1000100 PMC2707010

[21] Fazel S, Lichtenstein P, Grann M, Goodwin GM, Långström N (2010) Bipolar disorder and violent crime: New evidence from population-based longitudinal studies and systematic review. Arch Gen Psychiatry 67: 931–938. 20819987 10.1001/archgenpsychiatry.2010.97

[22] Swanson JW, Swartz MS, Van Dorn RA, Elbogen EB, Wagner HR, et al (2006) A national study of violent behavior in persons with schizophrenia. Arch Gen Psychiatry 63: 490–499. 16651506 10.1001/archpsyc.63.5.490

[23] Swanson J, Swartz M, Elbogen E (2004) Effectiveness of atypical antipsychotic medications in reducing violent behavior among persons with schizophrenia in community-based treatment. Schizophr Bull 30: 3–20. 15176758 10.1093/oxfordjournals.schbul.a007065

[24] Swanson JW, Van Dorn RA, Monahan J, Swartz MS (2006) Violence and leveraged community treatment for persons with mental disorders. Am J Psychiatry 163: 1404–1411. 16877654 10.1176/ajp.2006.163.8.1404

[25] Swanson JW, Swartz MS, Essock SM, Osher FC, Wagner HR, et al (2002) The social-environmental context of violent behavior in persons treated for severe mental illness. Am J Public Health 92: 1523–1531. 12197987 10.2105/ajph.92.9.1523 PMC1447272

[26] Wootton L, Buchanan A, Leese M, Tyrer P, Burns T, et al (2008) Violence in psychosis: Estimating the predictive validity of readily accessible clinical information in a community sample. Schizophr Res 101: 176–184. 18302982 10.1016/j.schres.2007.12.490

[27] Nolan KA, Volavka J, Czobor P, Sheitman B, Lindenmayer J-P, et al (2005) Aggression and psychopathology in treatment-resistant inpatients with schizophrenia and schizoaffective disorder. J Psychiatr Res 39: 109–115. 15504429 10.1016/j.jpsychires.2004.04.010

[28] Barkataki I, Kumari V, Das M, Taylor PJ, Sharma T (2006) Volumetric structural brain abnormalities in men with schizophrenia or antisocial personality disorder. Behav Brain Res 169: 239–247. 16466814 10.1016/j.bbr.2006.01.009

[29] Khan KS, Ter Riet G, Popay J, Nixon J, Kleijnen J (2001) Phase 5: Study quality assessment. In: Khan KS, Ter Riet G, Glanville J, Sowden AJ, Kleijnen J, editors. Undertaking systematic reviews of research on effectiveness: CRD’s guidance for carrying out or commissioning reviews. York: NHS Centre for Reviews and Dissemination, University of York.

[30] Chinn S (2000) A simple method for converting an odds ratio to effect size for use in meta-analysis. Stat Med 19: 3127–3131. 11113947 10.1002/1097-0258(20001130)19:22<3127::aid-sim784>3.0.co;2-m

[31] Rosenthal R, DiMatteo MR (2001) Meta-analysis: Recent developments in quantitative methods for literature review. Annu Rev Psychol 52: 59–82. 11148299 10.1146/annurev.psych.52.1.59

[32] Rosenberg MS (2010) A generalized formula for converting chi-square tests to effect sizes for meta-analysis. PLoS One 5: e10059. 20383281 10.1371/journal.pone.0010059 PMC2850938

[33] DeCoster J (2009) Meta-analysis notes. Available: http://www.stat-help.com/notes.html. Accessed 2012 June 1.

[34] Rosenthal JA (1996) Qualitative descriptors of strength of association and effect size. J Soc Serv Res 21: 37–59.

[35] Altman DG (1991) Some common problems in medical research. Practical Statistics for the Medical Sciences. London: Chapman & Hall/CRC.

[36] DerSimonian R, Laird N (1986) Meta-analysis in clinical trials. Control Clin Trials 7: 177–188. 3802833 10.1016/0197-2456(86)90046-2

[37] Higgins JPT, Thompson SG, Deeks JJ, Altman DG (2003) Measuring inconsistency in meta-analyses. BMJ 327: 557–563. 12958120 10.1136/bmj.327.7414.557 PMC192859

[38] Peters JL, Sutton AJ, Jones DR, Abrams KR, Rushton L (2006) Comparison of two methods to detect publication bias in meta-analysis. JAMA 295: 676–680. 16467236 10.1001/jama.295.6.676

[39] Egger M, Smith G, Schneider M, Minder C (1997) Bias in meta-analysis detected by a simple, graphical test. BMJ 315: 629–634. 9310563 10.1136/bmj.315.7109.629 PMC2127453

[40] Baker WL, White M, Cappelleri JC, Kluger J, Coleman CI (2009) Understanding heterogeneity in meta-analysis: The role of meta-regression. Int J Clin Pract 63: 1426–1434. 19769699 10.1111/j.1742-1241.2009.02168.x

[41] Kay SR, Fiszbein A, Opler LA (1987) The Positive and Negative Syndrome Scale (PANSS) for schizophrenia. Schizophr Bull 13: 261–276. 3616518 10.1093/schbul/13.2.261

[42] Wechsler D (2008) WAIS-IV Administration and Scoring Manual. San Antonio, TX: The Psychological Corporation.

[43] Wechsler D (2008) WAIS-IV Technical and Interpretive Manual. San Antonio, TX: The Psychological Corporation.

[44] Nelson HE (1982) National Adult Reading Test. Windsor, UK: NFER-Nelson.

[45] Heaton RK, Chelune GJ, Talley JL, Kay GG, Curtiss G (1993) Wisconsin Card Sorting Test manual. Odessa, FL: Psychological Assessment Resources.

[46] Douglas KS, Skeem JL (2005) Violence risk assessment: Getting specific about being dynamic. Psychol Pub Pol’y & Law 11: 347–383.

[47] Fazel S, Långström N, Hjern A, Grann M, Lichtenstein P (2009) Schizophrenia, substance abuse and violent crime. JAMA 301: 2016–2023. 19454640 10.1001/jama.2009.675 PMC4905518

[48] Bonta J, Law M, Hanson K (1998) The prediction of criminal and violent recidivism among mentally disordered offenders: A meta-analysis. Psychol Bull 123: 123–142. 9522681 10.1037/0033-2909.123.2.123

[49] Mullen PE (2006) Schizophrenia and violence: From correlations to preventive strategies. Advances in Psychiatric Treatment 12: 239–248.

[50] Swanson JW, Swartz MS, Van Dorn RA, Volavka J, Monahan J, et al (2008a) Comparison of antipsychotic medication effects on reducing violence in people with schizophrenia. Br J Psychiatry 193: 37–43. 18700216 10.1192/bjp.bp.107.042630 PMC2801826

[51] Topiwala A, Fazel S (2011) The pharmacological management of violence in schizophrenia: A structured review. Expert Review of Neurotheraputics 11: 53–63. 10.1586/ern.10.180 21158555

[52] Buckley P, Citrome L, Nichita C, Vitacco M (2011) Psychopharmacology of aggression in schizophrenia. Schizophr Bull 37: 930–936. 21860038 10.1093/schbul/sbr104 PMC3160216

[53] Zygmunt A, Olfson M, Boyer CA, Mechanic D (2002) Interventions to improve medication adherence in schizophrenia. Am J Psychiatry 159: 1653–1664. 12359668 10.1176/appi.ajp.159.10.1653

[54] Kooyman I, Dean K, Harvey S, Walsh E (2007) Outcomes of public concern in schizophrenia. Br J Psychiatry 191: s29–s36. 10.1192/bjp.191.50.s29 18019041

[55] Hiday VA, Swartz MS, Swanson JW, Borum R, Wagner HR (1999) Criminal victimization of persons with severe mental illness. Psychiatr Serv 50: 62–68. 9890581 10.1176/ps.50.1.62

[56] Larger M, Babidge N, Andrews D, Storey P, Nielssen O (2009) Major self-mutilation in the first episode of psychosis. Schizophr Bull 35: 1012–1021. 18495646 10.1093/schbul/sbn040 PMC2728813

[57] Webster CD, Martin ML, Brink J, Nicholls TL, Middleton C (2004) Short-Term Assessment of Risk and Treatability (START). Hamilton, ON: St Joseph’s Healthcare.

[58] Conner KR, Duberstein PR, Conwell Y, Caine ED (2002) Reactive aggression and suicide: Theory and evidence. Aggress Viol Behav 8: 413–432.

[59] Volavka J (2002) Neurobiology of violence. Washington, DC: American Psychiatric Publishing, Inc.

[60] Singh JP, Serper M, Reinharth J, Fazel S (2011) Structured assessment of violence risk in schizophrenia and other psychiatric disorders: A systematic review of the validity, reliability and item content of 10 available instruments. Schizophr Bull 37: 899–912. 21860036 10.1093/schbul/sbr093 PMC3160213

[61] Soyka M (2011) Neurobiology of aggression and violence in schizophrenia. Schizophr Bull 37: 913–920. 21860037 10.1093/schbul/sbr103 PMC3160223

[62] Yiend J, Chambers JC, Burns T, Doll H, Fazel S, et al (2011) Outcome measurement in forensic mental health research: An evaluation. Psychol Crime Law 17: 277–292.

[63] Allen NC, Bagade S, McQueen MB, Ioannidis JPA, Kavvoura FK (2008) Systematic meta-analyses and field synopsis of genetic association studies in schizophrenia: The SzGene database. Nat Genet 40: 827–834. 18583979 10.1038/ng.171

[64] Van Dorn RA, Desmarais SL, Singh JP (2011) Violence and victimization in adults with severe mental illness: Part 1 - An application of integrated data analysis. In: Needham I, Palmstierna T, Almvik R, Oud N, editors. Proceedings of the 7th European Congress on Violence in Clinical Psychiatry. Amsterdam, The Netherlands: Kavanah.

[65] Schwarzer G, Antes G, Schumacher M (2002) Inflation of type I error rate in two statistical tests for the detection of publication bias in meta-analyses with binary outcomes. Stat Med 21: 2465–2477. 12205693 10.1002/sim.1224

[66] Lancet (2010) Should protocols for observational research be registered? The Lancet 375: 348. 10.1016/S0140-6736(10)60148-1 20113809

[67] Farrington DP, Loeber R, Ttofi MM (2012) Risk and protective factors for offending. In: Welsh BC, Farrington DP, editors. The Oxford handbook of crime prevention. NY: Oxford University Press.

[68] Yu R, Geddes JR, Fazel S (2012) Personality disorders, violence and antisocial behavior: A systematic review and meta-regression analysis. J Personal Disord 26: 775–792. 10.1521/pedi.2012.26.5.775 23013345

[69] Singh JP, Grann M, Lichtenstein P, Långström N, Fazel S (2012) A novel approach to determining violence risk in schizophrenia: Developing a stepped strategy in 13,806 discharged patients. PLoS One 7: e31727. 22359622 10.1371/journal.pone.0031727 PMC3280996

[70] Burton A, Marougka S, Priebe S (2010) Do financial incentives increase treatment adherence in people with severe mental illness? A systematic review. Epidemiol Psichiatr Soc 19: 233–242. 21261219

[71] Wobrock T, Soyka M (2008) Pharmacotherapy of schizophrenia with comorbid substance use disorder - Reviewing the evidence and clinical recommendations. Prog Neuropsychopharmacol Biol Psychiatry 32: 1375–1385. 18394768 10.1016/j.pnpbp.2008.02.008

[72] Cipriani A, Pretty H, Hawton K, Geddes JR (2005) Lithium in the prevention of suicidal behavior and all-cause mortality in patients with mood disorders: A systematic review of randomized trials. Am J Psychiatry 162: 1805–1819. 16199826 10.1176/appi.ajp.162.10.1805

[73] Geddes JR (2005) Large simple trials in psychiatry: Providing reliable answers to important clinical questions. Epidemiol Psichiatr Soc 14: 122–126. 16255157 10.1017/s1121189x00006357

