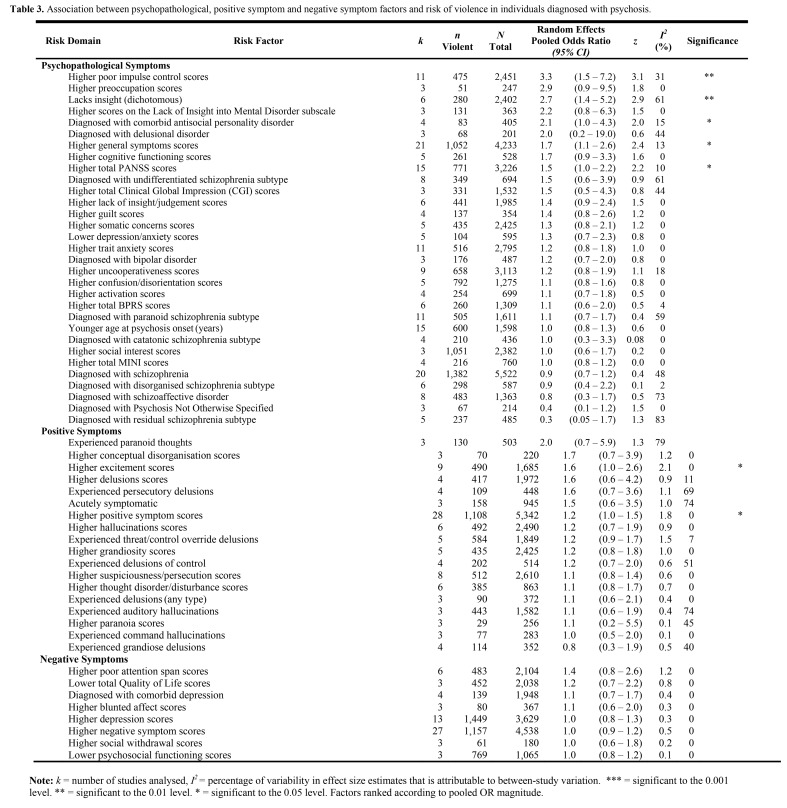# Correction: Risk Factors for Violence in Psychosis: Systematic Review and Meta-Regression Analysis of 110 Studies

**DOI:** 10.1371/annotation/f4abfc20-5a38-4dec-aa46-7d28018bbe38

**Published:** 2013-09-25

**Authors:** Katrina Witt, Richard van Dorn, Seena Fazel

There was a formatting error affecting the column alignment of Table 3. The corrected Table 3 can be found here: 

**Figure:**