# Supplementary material for: Risk Factors for Violence in Psychosis: Systematic Review and Meta-Regression Analysis of 110 Studies
Source: PLoS One. 2013 Feb 13;8(2):e55942. doi: 10.1371/journal.pone.0055942 (PMC3572179; doi:10.1371/journal.pone.0055942)
Supplement: Figure S1 — Full electronic search strategy for the CINAHL database depicting limits and Boolean key operators used as well as the number of “hits” for each search. (DOCX) [file pone.0055942.s001.docx]

**Full Electronic Search Strategy Utilised for CINAHL Database**

**(Limits: 1 January, 1982 – 31 December, 2011)**

|  |  | **Concept 1: Diagnosis** |  | **Concept 2: Violence** | **Number of “Hits”** | |
| --- | --- | --- | --- | --- | --- | --- |
|  |  |  | AND |  |  | |
| Text Words |  | “Schiz*” [Title/Abstract] |  | “Viol*” [Title/Abstract] | 140 |  |
|  | OR |  |  |  |  |  |
|  |  |  |  | “Crim*” [Title/Abstract] | 50 |  |
|  | OR |  |  |  |  |  |
|  |  |  |  | “Aggress*” [Title/Abstract] | 121 |  |
|  | OR |  |  |  |  |  |
|  |  |  |  | “Offend*” [Title/Abstract] | 23 |  |
|  | OR |  |  |  |  |  |
|  |  |  |  | “Danger*” [Title/Abstract] | 46 |  |
|  | OR |  |  |  |  |  |
|  |  |  |  | “Hosti*” [Title/Abstract] | 51 |  |
|  | AND |  |  |  |  |  |
|  |  | “Psych*” [Title/Abstract] |  | “Viol*” [Title/Abstract] | 2,272 |  |
|  | OR |  |  |  |  |  |
|  |  |  |  | “Crim*” [Title/Abstract] | 634 |  |
|  | OR |  |  |  |  |  |
|  |  |  |  | “Aggress*” [Title/Abstract] | 1,439 |  |
|  | OR |  |  |  |  |  |
|  |  |  |  | “Offend*” [Title/Abstract] | 374 |  |
|  | OR |  |  |  |  |  |
|  |  |  |  | “Danger*” [Title/Abstract] | 510 |  |
|  | OR |  |  |  |  |  |
|  |  |  |  | “Hosti*” [Title/Abstract] | 454 |  |
|  | AND |  |  |  |  |  |
|  |  | “Mental*” [Title/Abstract] |  | “Viol*” [Title/Abstract] | 1,457 |  |
|  | OR |  |  |  |  |  |
|  |  |  |  | “Crim*” [Title/Abstract] | 544 |  |
|  | OR |  |  |  |  |  |
|  |  |  |  | “Aggress*” [Title/Abstract] | 568 |  |
|  | OR |  |  |  |  |  |
|  |  |  |  | “Offend*” [Title/Abstract] | 308 |  |
|  | OR |  |  |  |  |  |
|  |  |  |  | “Danger*” [Title/Abstract] | 328 |  |
|  | OR |  |  |  |  |  |
|  |  |  |  | “Hosti*” [Title/Abstract] | 142 |  |
