# Supplementary material for: Risk Factors for Violence in Psychosis: Systematic Review and Meta-Regression Analysis of 110 Studies
Source: PLoS One. 2013 Feb 13;8(2):e55942. doi: 10.1371/journal.pone.0055942 (PMC3572179; doi:10.1371/journal.pone.0055942)
Supplement: Figure S2 — Flow-chart depicting the search strategy employed to locate the 110 studies included in the systematic review and meta-analysis. (DOC) [file pone.0055942.s002.doc]

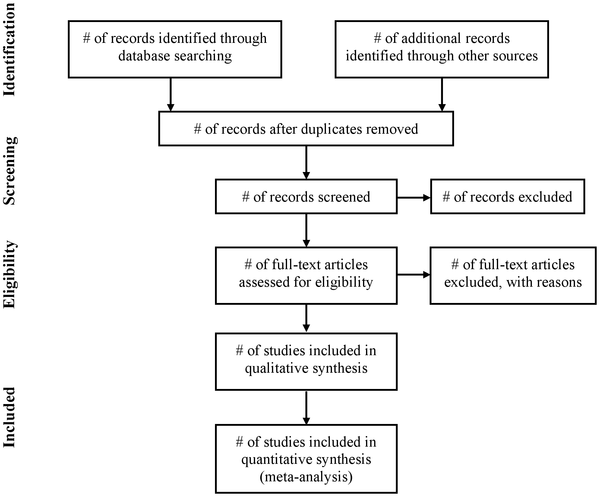


21 studies identified from reference lists

753 studies after duplicates removed

753 studies screened

410 studies excluded as not meeting inclusion criteria.

227 records removed as reviews, conference abstracts, letters, etc.

6 records removed as data

non-poolable owing to unique definition used.

110 studies included

157, 994 records identified through electronic databases

343 full-text studies evaluated for inclusion

227 studies excluded as not reporting primary data

6 studies excluded as included risk factors were not validated
