# Supplementary material for: Risk Factors for Violence in Psychosis: Systematic Review and Meta-Regression Analysis of 110 Studies
Source: PLoS One. 2013 Feb 13;8(2):e55942. doi: 10.1371/journal.pone.0055942 (PMC3572179; doi:10.1371/journal.pone.0055942)
Supplement: Table S1 — Association between risk factors replicated in only two primary studies and risk of violence in individuals diagnosed with psychosis. (DOCX) [file pone.0055942.s003.docx]

**Table S1.** Association between risk factors replicated in only two primary studies and risk of violence in individuals diagnosed with psychosis.

| **Risk Domain** | | **Risk Factor** | ***k*** | ***n***  **Violent** | | ***N***  **Total** | | **Random Effects**  **Pooled Odds Ratio**  ***(95% CI)*** | | ***z*** | ***I^2^***  **(%)** | **Significance** |
| --- | --- | --- | --- | --- | --- | --- | --- | --- | --- | --- | --- | --- |
| **Demographic** | | |  |  | |  | |  |  |  |  |  |
|  | History of non-violent victimization during adulthood | | 2 | 337 | 1,812 | | 2.8 | | (1.9 – 4.0) | 5.6 | 0 | *** |
|  | Immigrant | | 2 | 1,825 | 13,867 | | 2.7 | | (0.5 – 14.5) | 1.1 | 85^1^ |  |
|  | Less than 40 years of age at study enrolment | | 2 | 258 | 970 | | 2.6 | | (1.5 – 4.3) | 3.7 | 0 | *** |
|  | Unemployed and not in vocational training currently | | 2 | 53 | 83 | | 2.6 | | (0.7 – 10.0) | 1.4 | 0 |  |
|  | Older maternal age at birth (years) | | 2 | 135 | 271 | | 1.7 | | (0.4 – 7.4) | 0.8 | 30 |  |
|  | Failed to complete compulsory military service | | 2 | 63 | 121 | | 1.5 | | (0.6 – 3.6) | 0.9 | 0 |  |
|  | Lives in supported/sheltered accommodation currently | | 2 | 53 | 83 | | 1.0 | | (0.1 – 7.1) | 0.03 | 33 |  |
|  | Unemployed but in vocational training currently | | 2 | 53 | 83 | | 0.7 | | (0.2 – 2.4) | 0.5 | 0 |  |
| **Premorbid** | | |  |  |  | |  | |  |  |  |  |
|  | Higher premorbid adjustment in adulthood scores | | 2 | 55 | 126 | | 1.5 | | (0.6 – 3.9) | 0.9 | 0 |  |
|  | Parental history of drug misuse | | 2 | 26 | 320 | | 1.2 | | (0.6 – 2.2) | 0.6 | 0 |  |
|  | Did not live with both parents until the age of 16 | | 2 | 26 | 320 | | 1.1 | | (0.6 – 2.0) | 0.5 | 0 |  |
|  | Parental history of mental illness (any type) | | 2 | 26 | 320 | | 1.0 | | (0.6 – 1.4) | 0.0 | 0 |  |
|  | Higher total scores on the Premorbid Adjustment Scale | | 2 | 55 | 126 | | 1.0 | | (0.5 – 1.7) | 0.0 | 0 |  |
| **Criminal History** | | |  |  |  | |  | |  |  |  |  |
|  | Higher trait anger scores | | 2 | 11 | 149 | | 11.9 | | (1.3 – 106.5) | 2.2 | 0 | * |
|  | History of a criminal record (arrest/conviction) for any offence | | 2 | 170 | 732 | | 9.8 | | (0.3 – 261.3) | 1.3 | 71 |  |
|  | Higher physical aggression scores | | 2 | 104 | 206 | | 6.3 | | (0.8 – 47.8) | 1.7 | 11 |  |
|  | History of contact with police not resulting in arrest | | 2 | 126 | 325 | | 4.5 | | (1.1 – 18.7) | 2.1 | 0 | * |
|  | Higher antisocial behaviour scores | | 2 | 113 | 226 | | 2.1 | | (0.6 – 7.2) | 1.2 | 0 |  |
|  | Higher criticisms of others scores | | 2 | 98 | 196 | | 1.8 | | (0.6 – 5.4) | 1.1 | 0 |  |
|  | Recent violent ideations | | 2 | 60 | 312 | | 1.7 | | (0.2 – 12.9) | 0.5 | 34 |  |
|  | Higher projection of hostility scores | | 2 | 98 | 196 | | 1.6 | | (0.6 – 4.1) | 0.9 | 0 |  |
|  | History of violence against psychiatric staff | | 2 | 77 | 212 | | 1.3 | | (0.4 – 4.4) | 0.5 | 60 |  |
|  | Higher direction of hostility scores | | 2 | 98 | 196 | | 1.3 | | (0.6 – 2.6) | 0.7 | 0 |  |
|  | Higher psychopathic deceitful interpersonal style scores | | 2 | 44 | 194 | | 1.2 | | (0.4 – 3.1) | 0.3 | 27 |  |
|  | Higher psychopathic impulsive/irresponsible behaviour scores | | 2 | 44 | 194 | | 1.1 | | (0.7 – 1.6) | 0.4 | 0 |  |
|  | Greater number of prior convictions for violent offences | | 2 | 169 | 820 | | 1.0 | | (0.4 – 2.3) | 0.02 | 2 |  |
|  | Higher psychopathic deficient emotional experience scores | | 2 | 44 | 194 | | 1.0 | | (0.5 – 1.9) | 0.0 | 0 |  |
| **Psychopathological** | | |  |  |  | |  | |  |  |  |  |
|  | Poor family care during illness | | 2 | 63 | 212 | | 12.7 | | (3.8 – 42.2 | 4.1 | 0 | *** |
|  | Higher scores on the Lack of Insight into Consequences of Illness subscale | | 2 | 131 | 289 | | 4.8 | | (0.8 – 28.0) | 1.7 | 0 |  |
|  | Higher irritability scores | | 2 | 44 | 230 | | 4.1 | | (0.8 – 21.2) | 1.6 | 0 |  |
|  | Higher scores on the Lack of Insight into the Need for Treatment subscale | | 2 | 131 | 289 | | 3.8 | | (0.7 – 19.1) | 1.6 | 0 |  |
|  | Diagnosed with a comorbid personality disorder (any type) | | 2 | 258 | 970 | | 2.1 | | (1.5 – 2.9) | 4.3 | 0 | *** |
|  | Diagnosed with comorbid mania | | 2 | 207 | 655 | | 2.0 | | (0.8 – 5.0) | 1.4 | 73 |  |
|  | Higher tension scores | | 2 | 39 | 158 | | 1.9 | | (0.6 – 6.0) | 1.1 | 0 |  |
|  | Diagnosed with a comorbid psychiatric illness (any type) | | 2 | 53 | 83 | | 1.8 | | (0.2 – 12.5) | 0.6 | 0 |  |
|  | Onset of psychosis before 19 years of age | | 2 | 143 | 587 | | 1.8 | | (1.2 – 2.6) | 2.9 | 0 | ** |
|  | Lower psychoticism scores | | 2 | 755 | 876 | | 1.3 | | (0.1 – 15.8) | 0.2 | 61 |  |
|  | Acute illness onset | | 2 | 403 | 1,512 | | 1.1 | | (0.8 – 1.6) | 0.8 | 0 |  |
|  | Anxious behaviour during the study period | | 2 | 60 | 312 | | 1.0 | | (0.8 – 1.2) | 0.1 | 0 |  |
|  | Higher mannerisms/posturing scores | | 2 | 39 | 158 | | 1.0 | | (0.5 – 2.0) | 0.0 | 0 |  |
|  | Psychosocially stressed (at admission) | | 2 | 60 | 312 | | 1.0 | | (0.5 – 1.9) | 0.1 | 0 |  |
|  | Diagnosed with hebephrenic schizophrenia sub-type | | 2 | 40 | 82 | | 0.9 | | (0.2 – 3.5) | 0.05 | 10 |  |
|  | Insidious illness onset | | 2 | 229 | 661 | | 0.8 | | (0.6 – 1.2) | 0.8 | 0 |  |
| **Positive Symptoms** | | |  |  |  | |  | |  |  |  |  |
|  | Experienced hallucinations and delusions | | 2 | 64 | 233 | | 6.2 | | (2.1 – 18.4) | 3.3 | 0 | *** |
|  | Behaves bizarrely during the study period | | 2 | 30 | 241 | | 2.7 | | (0.3 – 26.0) | 0.8 | 90^1^ |  |
|  | Higher bizarre behaviour scores | | 2 | 80 | 160 | | 2.0 | | (0.6 – 6.7) | 1.2 | 0 |  |
|  | Experienced symptoms of mania | | 2 | 207 | 655 | | 1.6 | | (0.8 – 3.2) | 1.3 | 70 |  |
|  | Higher threat, control/override delusion scores | | 2 | 35 | 290 | | 1.3 | | (0.2 – 9.1) | 0.3 | 57 |  |
|  | Experienced hallucinations (any type) | | 2 | 60 | 312 | | 1.3 | | (0.7 – 2.5) | 0.9 | 0 |  |
|  | Experienced passivity delusions | | 2 | 66 | 228 | | 1.2 | | (0.1 – 9.8) | 0.2 | 64 |  |
|  | Experienced jealousy delusions | | 2 | 44 | 222 | | 0.9 | | (0.5 – 1.6) | 0.1 | 0 |  |
|  | Experienced somatic delusions | | 2 | 44 | 222 | | 0.9 | | (0.3 – 2.1) | 0.2 | 13 |  |
| **Negative Symptoms** | | |  |  |  | |  | |  |  |  |  |
|  | Poor self care | | 2 | 60 | 312 | | 1.9 | | (0.3 – 11.7) | 0.7 | 82^1^ |  |
|  | Higher poor rapport scores | | 2 | 36 | 163 | | 1.9 | | (0.6 – 5.9) | 1.1 | 0 |  |
|  | Higher avolition scores | | 2 | 80 | 160 | | 1.8 | | (0.6 – 5.6) | 1.1 | 0 |  |
|  | Higher alogia scores | | 2 | 80 | 160 | | 1.4 | | (0.6 – 3.4) | 0.8 | 0 |  |
|  | Lower anergia scores | | 2 | 57 | 285 | | 1.0 | | (0.7 – 1.6) | 0.4 | 0 |  |
| **Neuropsychological** | | |  |  |  | |  | |  |  |  |  |
|  | Smaller total brain volume (cm^3^) | | 2 | 35 | 69 | | 2.5 | | (0.6 – 9.6) | 1.3 | 0 |  |
|  | Higher executive functioning scores | | 2 | 35 | 155 | | 1.8 | | (0.6 – 5.4) | 1.1 | 0 |  |
|  | Higher block design scores | | 2 | 39 | 82 | | 1.3 | | (0.04 – 43.4) | 0.1 | 72 |  |
|  | Higher digit symbol scores | | 2 | 39 | 82 | | 1.2 | | (0.05 – 26.9) | 0.1 | 68 |  |
|  | Diagnosed with a comorbid neurological illness (any type) | | 2 | 53 | 83 | | 1.1 | | (0.3 – 3.9) | 0.2 | 0 |  |
|  | Higher object assembly scores | | 2 | 39 | 82 | | 1.1 | | (0.1 – 13.2) | 0.1 | 59 |  |
|  | Higher picture arrangement scores | | 2 | 39 | 82 | | 1.1 | | (0.05 – 21.2) | 0.06 | 67 |  |
|  | Lower total Stroop test scores | | 2 | 47 | 85 | | 1.0 | | (0.3 – 2.6) | 0.04 | 8 |  |
| **Substance Misuse** | | |  |  |  | |  | |  |  |  |  |
|  | Diagnosed with comorbid alcohol use disorder | | 2 | 24 | 272 | | 1.5 | | (0.5 – 4.0) | 0.8 | 0 |  |
|  | Diagnosed with comorbid drug use disorder | | 2 | 24 | 272 | | 1.5 | | (0.5 – 4.0) | 0.8 | 0 |  |
|  | Recent tobacco use | | 2 | 91 | 995 | | 1.3 | | (0.4 – 3.7) | 0.5 | 55 |  |
|  | History of cocaine misuse | | 2 | 43 | 220 | | 1.1 | | (0.5 – 2.4) | 0.2 | 0 |  |
| **Treatment Related** | | |  |  |  | |  | |  |  |  |  |
|  | Prescribed antiparkinsonism medication | | 2 | 82 | 222 | | 1.7 | | (0.9 – 3.2) | 1.7 | 0 |  |
|  | Prescribed clozapine (rather than any other antipsychotic agent) | | 2 | 20 | 150 | | 1.4 | | (0.5 – 3.9) | 0.7 | 0 |  |
|  | Longer duration of previous inpatient admissions (months) | | 2 | 220 | 2,263 | | 1.2 | | (0.6 – 2.1) | 0.6 | 0 |  |
|  | Longer cumulative duration of inpatient psychiatric treatment (months) | | 2 | 33 | 144 | | 1.0 | | (0.3 – 3.2) | 0.08 | 18 |  |
| **Suicidality** | | |  |  |  | |  | |  |  |  |  |
|  | Higher self-criticism scores | | 2 | 98 | 196 | | 1.8 | | (0.6 – 5.6) | 1.1 | 0 |  |
|  | Recent self-harm | | 2 | 241 | 647 | | 1.6 | | (0.1 – 15.8) | 0.4 | 84^1^ |  |
|  | Higher suicidality scores | | 2 | 19 | 126 | | 1.0 | | (0.5 – 1.8) | 0.0 | 0 |  |
|  | Recent suicidal ideations | | 2 | 60 | 312 | | 0.9 | | (0.5 – 1.7) | 0.1 | 0 |  |

**Note:** *k* = number of studies analyzed, *I^2^* = percentage of variability in effect size estimates that is attributable to between-study variation. *** = significant to the 0.001 level.

** = significant to the 0.01 level. * = significant to the 0.05 level. Factors ranked within risk domains according to pooled OR magnitude.

^1^ No study characteristic investigated was significantly associated with heterogeneity.
