# Supplementary material for: Risk Factors for Violence in Psychosis: Systematic Review and Meta-Regression Analysis of 110 Studies
Source: PLoS One. 2013 Feb 13;8(2):e55942. doi: 10.1371/journal.pone.0055942 (PMC3572179; doi:10.1371/journal.pone.0055942)
Supplement: Table S2 — Methodological summary and reference list for the110 studies included in this systematic review and meta-analysis. (DOCX) [file pone.0055942.s004.docx]

**Table S2.** Methodological summary and reference list for the110 studies included in this systematic review and meta-analysis.

| **Reference** | **Country** | **Participant Details** | **Violence Outcome Measure** | **Diagnostic Criteria** | **Diagnostic Screening Tool** | **Diagnosis** | **Violent**  **(*n*)** | **Total**  **(*N*)** |
| --- | --- | --- | --- | --- | --- | --- | --- | --- |
| **Abu-Akel, 2004** [1] | Israel | **Participants:** males, between 20 and 54 years of age, meeting criteria for a diagnosis of paranoid schizophrenia, treated as inpatients in an open psychiatric ward (non-violent group) or in a maximum-security psychiatric ward (violent group).  **Exclusion Criteria:** females, individuals diagnosed with schizophrenia subtypes other than paranoid. | History of a minimum of three assaults. | ICD-10 | None Stated | Paranoid schizophrenia | 12 | 24 |
| **Abushua'lea, 2006** **[**2] | Israel | **Participants:** males, between 20 and 54 years of age, meeting criteria for a diagnosis of schizophrenia, treated as inpatients in an open psychiatric ward (non-violent group) or in a maximum-security psychiatric ward (violent group).  **Exclusion Criteria:** females. | History of a minimum of three assaults. | ICD-10 | None Stated | Schizophrenia | 19 | 35 |
| **Arango, 1999** [3] | Spain | **Participants:** males and females, 18 years of age or older, meeting criteria for schizophrenia or schizoaffective disorder, consecutively a treated as an inpatient in a short-term psychiatric unit within a university general hospital for a minimum of 3 days between May to September, 1996.  **Exclusion criteria:** individuals who refused to participate, were transferred to another hospital during the study period, or were discharged within 72 hours of admission | An incident of physical aggression against others scoring three or more on the Overt Aggression Scale (OAS) during the admission period. | DSM-IV | None Stated | Schizophrenia or  schizoaffective disorder | 16 | 63 |
| **Arango, 2006** [4] | Spain | **Participants:** males and females, 18 years of age or older, meeting criteria for schizophrenia, treated as an inpatient in one of three general or psychiatric hospitals, had an incident of violence which scored three or more on the physical aggression sub-scale of the Modified Overt Aggression Scale (MOAS) in the one year preceding study enrolment.  **Exclusion Criteria:** individuals diagnosed with comorbid axis I disorders, alcohol or drug abuse or dependence, or mental retardation. | An incident of violence scoring three or more on the physical aggression sub-scale of the MOAS during the one year follow-up period post-dating study enrolment. | DSM-IV | None Stated | Schizophrenia | 20 | 46 |
| **Barkataki, 2005** [5] | UK | **Participants:** males, between 18 and 45 years of age, meeting criteria for schizophrenia, treated as an inpatient in a general psychiatric facility (non-violent group) or incarcerated in a high-security forensic psychiatric hospital or a medium-security forensic psychiatric ward (violent group).  **Exclusion Criteria:** females, left-handers, individuals whose first language is a language other than English, individuals currently abusing illicit drugs, or with a history of neurological conditions (e.g., epilepsy), head injury, or comorbid antisocial personality disorder. | An incident of violence scoring four or more on the Gunn and Robertson Scale for Violence, indicative of a fatal or near-fatal attack on the victim. | DSM-IV | SCID | Schizophrenia | 13 | 28 |
| **Barkataki, 2006** [6] | UK | **Participants:** males, between 18 and 45 years of age, meeting criteria for schizophrenia, treated in an open psychiatric facility (non-violent group) or incarcerated in a high-security forensic psychiatric hospital or a medium-security forensic psychiatric ward (violent group).  **Exclusion Criteria:** females, left-handers, individuals whose first language is a language other than English, individuals currently abusing, or have abused in the last two years, illicit drugs, or with a history of neurological conditions (e.g., epilepsy), head injury, or comorbid antisocial personality disorder. | An incident of violence which scoring four or more on the Gunn and Robertson Scale for Violence, indicative of a fatal or near-fatal attack on the victim. | DSM-IV | SCID | Schizophrenia | 13 | 28 |
| **Bartels, 1991** [7] | USA | **Participants:** males and females, 18 years of age or older, meeting criteria for schizophrenia or schizoaffective disorder, treated as an outpatient by the ambulatory community service team between November to December, 1983.  **Exclusion Criteria:** None stated. | An incident of severe or extremely severe violence according to an idiosyncratic 5-point hostility measure developed by the study’s authors from the 7-point hostility item of the Brief Psychiatric Rating Scale (BPRS) over the one year follow-up period, indicative of destruction of property, assault not occasioning harm to the victim, assault causing actual harm to the victim or assault with the potential to have caused harm to the victim. | DSM-III | None Stated | Schizophrenia or schizoaffective Disorder | 17^[[1]](#endnote-1)^ | 81^[[2]](#endnote-2)^ |
| **Belfrage, 1998** [8] | Sweden | **Participants:** males and females, between 17 and 70 years of age, meeting criteria for schizophrenia, affective psychosis or paranoia, discharged from inpatient care at one of three mental hospitals in 1986 and followed up in the community ten years later.  **Exclusion Criteria:** individuals who died during the ten year follow-up period. | Sentenced for a violent criminal offence during the 10 year follow-up period. | ICD-9 | None Stated | Schizophrenia, affective psychoses or paranoia | 183 | 893 |
| **Bitter, 2005** [9] | IC^[[3]](#endnote-3)^ | **Participants:** males and females, 18 years of age or older, meeting criteria for schizophrenia, treated as an outpatient in any psychiatric centre of the 27 participating countries, prescribed monotherapy with either clozapine, olanzapine, risperidone, quetiapine or haloperidol.  **Exclusion criteria:** individuals with no baseline information available, those not prescribed antipsychotic therapy, prescribed to poly-antipsychotic therapy, prescribed to an antipsychotic other than clozapine, olanzapine, risperidone, quetiapine or haloperidol, switched to a different antipsychotic half-way through assessment period, incomplete information regarding antipsychotic dosage, with incomplete information regarding hostility or with no six month follow-up information available. | Answering in the affirmative to the following question: “Has the patient exhibited verbal or physical hostility or aggression over the follow-up period?” | ICD-10  or  DSM-IV | None Stated | Schizophrenia | 362^[[4]](#endnote-4)^ | 3135 |
| **Bobes, 2009** [10] | Spain | **Participants:** males and females, 18 years of age or older, meeting criteria for schizophrenia, consecutively treated as an outpatient between April to December, 2005.  **Exclusion Criteria:** individuals recently diagnosed with schizophrenia, diagnosed with a mental illness other than schizophrenia, prescribed antipsychotic medication for less than three months, or who refused to participate | An incident of aggressiveness in the week preceding study enrolment scoring three or more on any sub-scale of the MOAS. | DSM-IV-TR | None Stated | Schizophrenia | 41 | 895 |
| **Buckley, 2004** [11] | USA | **Participants:** males and females, 18 years of age or older, meeting criteria for schizophrenia or schizoaffective disorder, treated in one of two community mental health centres (non-violent group) or incarcerated in a county jail (violent group).  **Exclusion Criteria:** none stated. | An incident of physical aggression against property or persons for which criminal charges have been laid. | DSM-IV | None Stated | Schizophrenia or schizoaffective disorder | 115 | 226 |
| **Calcedo-Barba, 1994** [12] | Spain | **Participants:** males and females, 18 years of age or older, meeting criteria for schizophrenia (paranoid sub-type), admitted to the psychiatry department of a university hospital due to relapse (non-violent group) or a violent incident (violent group).  **Exclusion Criteria:** individuals with no collateral informant who could provide information on the participant’s behaviour in the community. | An incident of violence in the week preceding study enrolment scoring two or more on the physical violence sub-scale of the OAS. | DSM-III-R | None Stated | Paranoid sub-type schizophrenia | 19 | 58 |
| **Cannon, 2002** [13] | Finland | **Participants:** males and females born between 1951 and 1960 in Helsinki, meeting criteria for schizophrenia, schizoaffective disorder or schizophreniform disorder.  **Exclusion Criteria:** none stated. | Convicted of a violent crime including: homicide, assault, robbery, arson, illegal possession of a weapon, sexual offences and domestic violence up until 1995. | ICD-8, or  ICD-9 | None Stated | Schizophrenia, schizoaffective disorder or schizophreniform disorder | 36^[[5]](#endnote-5)^ | 636 |
| **Chang, 2011** [14] | China | **Participants:** males and females, between 15 and 25 years of age, meeting criteria for first-onset schizophrenia, consecutively treated as an outpatient between July 2001 and August 2003.  **Exclusion Criteria:** individuals diagnosed with mental retardation, psychosis due to a medical condition, substance induced psychosis, patients with more than one month of treatment prior to study enrolment and individuals with incomplete or missing medical files. | History of violence as revealed by clinical or forensic records. | ICD-10 | None Stated | Schizophrenia | 52 | 461^[[6]](#endnote-6)^ |
| **Cheung, 1996** [15] | Australia | **Participants:** males and females, 18 years of age and older, meeting criteria for schizophrenia, treated as an inpatient within a rehabilitation ward.  **Exclusion Criteria:** individuals not currently stabilised on medication, not currently experiencing at least one positive symptom, with a current diagnosis of substance abuse, a history of head injury, intellectual impairment, or those unable to communicate effectively in English. | Two or more incidents of physical aggression towards objects or persons during a ward survey conducted prior to study enrolment, and at least one incident of physical aggression towards objects or persons each month between the conclusion of this ward survey and the commencement of the study. | DSM-III-R | SCID | Schizophrenia | 31 | 62 |
| **Cheung, 1997a** [16] | Australia | **Participants:** males and females, 18 years of age or older, meeting criteria for schizophrenia, treated as an inpatient within a rehabilitation ward.  **Exclusion Criteria:** individuals not currently stabilised on medication, not currently experiencing at least one positive symptom, with a current diagnosis of substance abuse, a history of head injury, intellectual impairment, or those unable to communicate effectively in English. | Two or more incidents of physical aggression towards objects or persons during a ward survey conducted prior to study enrolment, and at least one incident of physical aggression towards objects or persons each month between the conclusion of this ward survey and the commencement of the study. | DSM-III-R | SCID | Schizophrenia | 31 | 62 |
| **Cheung, 1997b** [17] | Australia | **Participants:** males and females, 18 years of age or older, meeting criteria for schizophrenia, treated as an inpatient within a rehabilitation ward.  **Exclusion Criteria:** individuals not currently stabilised on medication, not currently experiencing at least one positive symptom, with a current diagnosis of substance abuse, a history of head injury, intellectual impairment, those unable to communicate effectively in English, or those whose urine test was positive for illicit substances. | Two or more incidents of physical aggression towards objects or persons during a ward survey conducted prior to study enrolment, and at least one incident of physical aggression towards objects or persons each month between the conclusion of this ward survey and the commencement of the study. | DSM-III-R | SCID | Schizophrenia | 31 | 62 |
| **Cheung, 1997c** [18] | Australia | **Participants:** males and females, 18 years of age or older, meeting criteria for schizophrenia, treated as an inpatient within a rehabilitation ward.  **Exclusion Criteria:** individuals not currently stabilised on medication, not currently experiencing at least one positive symptom, with a current diagnosis of substance abuse, a history of head injury, intellectual impairment, those unable to communicate effectively in English, or those whose urine test was positive for illicit substances. | Two or more incidents of physical aggression observed using the Staff Observation Aggression Scale (SOAS) during an eight week screening survey, and at least one incident of physical aggression towards objects or persons each month between the conclusion of this survey and the commencement of the study. | DSM-III-R | SCID | Schizophrenia | 31 | 62 |
| **Chung, 2006** [19] | South Korea | **Participants:** males, between 18 and 65 years of age, meeting criteria for schizophrenia or schizoaffective disorder, treated as inpatients in a general psychiatric hospital (non-violent group) or a forensic psychiatric hospital (violent group).  **Exclusion Criteria:** females, individuals diagnosed with physical or neurological abnormalities, a history of organic brain damage, or those unable to communicate effectively in Korean. | Convicted of homicide. | DSM-IV | None Stated | Schizophrenia or schizoaffective disorder | 93 | 186 |
| **Chung, 2010** [20] | South Korea | **Participants:** males, between 18 and 65 years of age, meeting criteria for schizophrenia, treated as inpatients in a general psychiatric hospital (non-violent group) or a forensic psychiatric hospital (violent group).  **Exclusion Criteria:** females, left-handers, individuals diagnosed with a physical or neurological abnormalities, mental retardation, with a history of organic brain damage, those unable to communicate effectively in Korean, or individuals who have not been symptomatically stabilized with stable doses of antipsychotic medication for a minimum of two months prior to study enrolment. | Convicted of homicide. | DSM-IV | SCID | Schizophrenia | 51 | 101 |
| **Cuffel, 1994** [21] | USA | **Participants:** males and females, between 18 and 55 years of age, meeting criteria for schizophrenia, or schizoaffective disorder, treated as an outpatient by a general hospital research clinic between 1985 and 1989, participating in a randomised controlled trial of fluphenazine decanoate in one of three dosages.  **Exclusion Criteria:** individuals diagnosed with a substance or alcohol dependence, or those with an extensive legal history. | An incident of verbal threats to harm others, non-verbal threats to harm others, throwing objects at others, physical assaults, use of a weapon, arson and damaging property in either months one to three of the trial, or, in months three to six of the trial. | DSM-III-R | SCID | Schizophrenia or schizoaffective disorder | 13 | 89^[[7]](#endnote-7)^ |
| **Dean, 2006** [22] | UK | **Participants:** females, between 18 and 65 years of age, hospitalised on at least two separate occasions for psychotic symptoms, meeting criteria for schizoaffective disorder, schizophrenia, psychosis not otherwise specified or bipolar disorder, participating in a randomised controlled trial of intensive community case management at one of four urban mental health services, and followed for two years whilst living in the community.  **Exclusion Criteria:** males, individuals diagnosed with an organic brain disorder, or with a history of substance misuse. | An incident of actual physical assault over the two year follow-up period. | ICD-10 | OPCRIT | Schizophrenia, schizoaffective disorder, psychosis not otherwise specified or bipolar disorder. | 53 | 304 |
| **Dean, 2007** [23] | UK | **Participants:** males and females, between 16 and 64 years of age, meeting criteria for a psychotic disorder, treated at a first-episode psychosis centre between September 1997 and August 1999.  **Exclusion Criteria:** individuals diagnosed with a mental illness other than psychosis. | Precipitating reason for treatment was due to an incident of a violent or hazardous act or an incident of behaviour perceived as threatening to others. Alternatively, a documented history of violence or aggression, including: physical assault, use of a weapon, arson, extensive property damage, verbally threatening others, delusional content related to thoughts of wanting to harm others, or, behaving in an aggressive or hostile manner towards others was required. | ICD-10 | RDC | Various psychotic disorders | 194 | 495 |
| **Ellouze, 2009** [24] | Tunisia | **Participants:** males and females, 18 years of age or older, meeting criteria for schizophrenia, treated as inpatients in a general hospital between September to November, 2006.  **Exclusion criteria:** individuals who had committed homicide, attempted homicide, armed offences, sexual offences or self-harm. | At least one incident of physical aggression directed against others during the one year prior to study enrolment. | DSM-IV | None Stated | Schizophrenia | 30 | 60 |
| **Eriksson, 2010** [25] | Sweden | **Participants:** males, born between 1949 and 1951 and conscripted into the Swedish Army between July 1969 and June 1970, discharged from hospital with a diagnosis of schizophrenia or schizoaffective disorder on at least one occasion, followed up in 2005.  **Exclusion Criteria:** individuals who could not be conscripted into the Swedish Army due to intellectual handicap, psychiatric illness or imprisonment. | Convicted of actual or attempted homicide, manslaughter, assault, robbery, assaulting or threatening a police officer, forcible confinement or sexual assault. | ICD-7, -8, -9 or -10. | None Stated | Schizophrenia or schizoaffective disorder, | 93^[[8]](#endnote-8)^ | 264 |
| **Erkiran, 2006** [26] | Turkey | **Participants:** males and females, between 18 and 59 years of age, meeting criteria for schizophrenia, schizoaffective disorder, schizophreniform disorder or delusional disorder either with or without comorbid substance use disorder, treated as an inpatient in a general psychiatric hospital.  **Exclusion Criteria:** none stated. | An incident of violence in the three days preceding study enrolment scoring two or more on the physical violence sub-scale of the OAS. | DSM-IV | None Stated | Schizophrenia spectrum disorder | 22 | 133 |
| **Fazel, 2009** [27] | Sweden | **Participants:** males and females, 15 years of age or older, discharged from hospitals between January 1973 and December, 2004 on at least two separate occasions with a diagnosis of schizophrenia, followed in the community until emigration, death or conviction for a violent offence.  **Exclusion Criteria:** individuals whose diagnosis of schizophrenia made on less than two separate occasions. | Conviction for a violent offence, including: homicide, attempted homicide, aggravated assault, common assault, robbery, arson, any sexual offence, sexual harassment, illegal threats and intimidation. | ICD-8, -9  or -10 | None Stated | Schizophrenia | 1,792 | 13,806 |
| **Fazel, 2010** [28] | Sweden | **Participants:** males and females, 15 years of age or older, discharged from hospitals between 1988 and 2001 on at least two separate occasions with a diagnosis of schizophrenia, followed in the community for six months post-discharge.  **Exclusion Criteria:** participants convicted of infanticide or a violent offence other than homicide. | Convicted of homicide, including: actual and attempted murder and manslaughter. | ICD-9 or -10 | None Stated | Schizophrenia | 47 | 152 |
| **Flannery, 1998** [29] | USA | **Participants:** males and females, 18 years of age or older, meeting criteria for various schizophrenia spectrum disorders, inpatients involuntarily committed to a government mental health facility.  **Exclusion Criteria:** individuals with private medical insurance. | One or more positive ratings on a checklist adapted from the New York State Level or Care Rating Scale, including the items: history of attempted murder, assault, sexually assault, molestation of children, arson, use of a weapon, dangerous behaviour, property destruction and threats to kill. | DSM-III-R | None Stated | Schizophrenia  spectrum disorder | 737 | 847 |
| **Foley, 2005** [30] | Republic of Ireland | **Participants:** males and females, between 16 and 65 years of age, meeting criteria for schizophrenia, delusional disorder, substance induced psychosis, psychotic depression, organic psychosis, psychosis NOS and bipolar disorder, treated as an inpatient or outpatient.  **Exclusion Criteria:** none stated. | A score of greater than one on any of the following MOAS sub-scales: aggression against property, autoaggression, or, aggression against others. Additionally, participants must have committed at least one incident of physical violence including throwing an object, harming themselves or physically attacking others. | DSM-III-R | None Stated | Schizophrenia, delusional disorder, substance induced psychosis, psychotic depression, organic psychosis, psychosis NOS or bipolar disorder | 25^[[9]](#endnote-9)^ | 157 |
| **Fresan, 2004** [31] | Mexico | **Participants:** males and females, between 17 and 45 years of age, meeting criteria for schizophrenia, treated as an outpatient.  **Exclusion Criteria:** individuals with comorbid alcohol or substance abuse in the six months prior to study enrolment. | Incidents in the week preceding study enrolment which together scored seven or more on the total aggression sub-scale of the OAS. | DSM-IV | SCID | Schizophrenia | 27 | 75 |
| **Fresan, 2005** [32] | Mexico | **Participants:** males and females, between 18 and 50 years of age, meeting criteria for schizophrenia, currently experiencing an acute episode of psychosis, treated as an inpatient or outpatient.  **Exclusion Criteria:** individuals with comorbid alcohol or substance abuse in the six months prior to study enrolment. | Incidents in the week preceding study enrolment which together scored seven or more on the total aggression sub-scale of the OAS. | DSM-IV | SCID | Schizophrenia | 20 | 100 |
| **Fresan, 2007a** [33] | Mexico | **Participants:** males and females, between 17and 45 years of age, meeting criteria for schizophrenia, treated as an outpatient.  **Exclusion Criteria:** individuals with comorbid alcohol or substance abuse in the six months prior to study enrolment. | Incidents in the week preceding study enrolment which together scored seven or more on the total aggression sub-scale of the OAS. | DSM-IV | SCID | Schizophrenia | 61 | 102 |
| **Fresan, 2007b** [34] | Mexico | **Participants:** males and females, between 17 and 50 years of age, meeting criteria for schizophrenia, currently experiencing an acute episode of psychosis, not received oral antipsychotic medication for at least four weeks prior to study enrolment or depot antipsychotic for at least six months prior to study enrolment, and treated as outpatients or inpatients.  **Exclusion Criteria:** individuals with axis I comorbidity, a history of bipolar disorder, medical or neurological illnesses, hearing loss, current substance abuse or a history of substance dependence within the previous 6 months. | Participants’ usual pattern of behaviour in the month preceding study enrolment rated as seven or more on the total aggression sub-scale of the OAS. | DSM-IV | SCID | Schizophrenia | 14 | 32 |
| **Fullam, 2008** [35] | UK | **Participants:** males, 18 years of age or older, meeting criteria for schizophrenia, inpatients in medium and high security forensic psychiatric hospitals.  **Exclusion Criteria:** individuals with a history of organic brain disorder, head injury, a recent history of receiving electroconvulsive shock therapy, those not currently symptomatically stable on medication, or those unable to provide informed consent. | One or more incidents of physical aggression since admission in which the participant was the clear instigator or co-instigator of the incident. | DSM-IV | None Stated | Schizophrenia | 33 | 82 |
| **Guy, 2003** [36] | UK | **Participants:** males, between 18 and 35 years of age, meeting criteria for schizophrenia, inpatients in the psychiatric department of a general hospital (non-violent group) or a maximum security forensic psychiatric institution (violent group).  **Exclusion Criteria:** individuals too unwell to understand study procedure, and those who refused consent to participate. | Charged with actual bodily harm against another, regardless of severity of injury to the victim. | ICD-10 | None Stated | Schizophrenia | 20 | 40 |
| **Hatta, 1999** [37] | Japan | **Participants:** males, between 18 and 64 years of age, meeting criteria for schizophrenia, delusional disorder or schizotypal disorder, inpatients in a psychiatric intensive care unit, prescribed parenteral benzodiazepines due to non-compliance with oral antipsychotic medications.  **Exclusion Criteria:** individuals medicated in the year prior to admission or those diagnosed with comorbid alcohol or substance misuse. | A score on the verbal aggression sub-scale of the OAS of three or more indicative of use of threatening language towards others. | ICD-10 | None Stated | Schizophrenia, delusional disorder or schizotypal disorder | 21^[[10]](#endnote-10)^ | 47 |
| **Herrera, 1988** [38] | USA | **Participants:** males, between 25 and 44 years of age, meeting criteria for schizophrenia, treated as inpatients, previously resistant to antipsychotic treatment with at least three different antipsychotics over two and a half years prior to study enrolment, currently prescribed high-potency antipsychotic medication.  **Exclusion Criteria:** individuals not experiencing an acute episode of psychosis, or those with a history of organic brain disorder, mental retardation, comorbid alcohol or substance abuse in the two weeks prior to study enrolment, or a physical illness. | A score of three or greater on Lion’s Scale of Inpatient Violence (LSIV). | DSM-III | None Stated | Schizophrenia | 7 | 16 |
| **Hodgins, 2003** [39] | IC | **Participants:** males, 18 years of age or older, meeting criteria for schizophrenia or schizoaffective disorder, recently discharged from one of four general psychiatric hospitals (non-violent group) or one of four forensic psychiatric hospitals (violent group) and assessed for violence in the community at six months post-discharge.  **Exclusion Criteria:** females. | At least one incident of the following behaviours committed during the six month observation period: throwing objects at others, pushing, shoving, grabbing, slapping, kicking, biting, choking or hitting another, rape, and using a weapon in a threatening manner. | DSM | SCID | Schizophrenia or schizoaffective disorder | 11 | 112^[[11]](#endnote-11)^ |
| **Hodgins, 2011** [40] | IC | **Participants:** males and females, 18 years of age or older, meeting criteria for schizophrenia or schizoaffective disorder, recently discharged from one of four general psychiatric hospitals (non-violent group) or one of four forensic psychiatric hospitals (violent group) and now living in the community.  **Exclusion Criteria:** none stated. | At least one incident of the following behaviours committed during the six month observation period: throwing objects at others, pushing, shoving, grabbing, slapping, kicking, biting, choking or hitting another, rape, and using a weapon in a threatening manner. | DSM-IV | SCID | Schizophrenia, schizoaffective disorder or schizophreniform disorder | 51 | 251 |
| **Hong, 2008** [41] | South Korea | **Participants:** males, between 18 and 65 years of age, meeting criteria for schizophrenia, treated as inpatients in one of two general psychiatric hospitals (non-violent group) or in a forensic psychiatric hospital (violent group), currently symptomatically stabilised and on stable doses of antipsychotic medication within the two months prior to study enrolment.  **Exclusion Criteria:** females, individuals not symptomatically stabilised or have not been prescribed stable doses of antipsychotic medications for at least the two months prior to study enrolment, or those with a history of head trauma, diagnosed with a neurological problem or with serious medical problems. | Convicted of homicide. | DSM-IV | SCID | Schizophrenia | 93 | 193 |
| **Hoptman, 1999** [42] | USA | **Participants:** males, 18 years of age or older, meeting criteria for a major mental illness (97% met criteria for schizophrenia), treated as an inpatient in a maximum security forensic psychiatric hospital between October, 1991 and April, 1996.  **Exclusion Criteria:** females. | An incident of at least one assault during the 12 week follow-up period. | DSM-III-R  . | SCID | 97% of sample diagnosed with schizophrenia | 60^[[12]](#endnote-12)^ | 183 |
| **Iancu, 2010** [43] | Israel | **Participants:** males, 18 years of age or older, meeting criteria for schizophrenia, treated as an inpatient in a mental health clinic.  Exclusion Criteria: females. | Total scores on the OAS. | DSM-IV | SCID | Schizophrenia |  | 68^[[13]](#endnote-13)^ |
| **Jones, 2001** [44] | UK | **Participants:** males and females, 18 years of age or older, meeting criteria for schizophrenia, treated as an outpatient or inpatient.  **Exclusion Criteria:** females, individuals of non-white ethnicity, immigrants, or those whose parents were born outside of the UK or Republic of Ireland. | Total score on the OAS of 13 or greater, indicative of threatening behaviour, striking, kicking, pushing, and pulling the hair of others, attacking another causing mild injury, and attacking another causing severe physical injury. | DSM-IV | SCAN | Schizophrenia | 68 | 180 |
| **Kim, 2009** [45] | South Korea | **Participants:** males and females, 18 years of age or older, meeting criteria for schizophrenia, treated as an inpatient at one of two psychiatric hospitals between 2004 and 2007.  **Exclusion Criteria:** individuals with comorbid axis I psychiatric disorders. | At least two incidents of physical violence requiring seclusion in the two weeks preceding study enrolment in addition to a history of at least two serious assaults committed at any point. | DSM-IV | None Stated | Schizophrenia | 46 | 103 |
| **Koen, 2004** [46] | South Africa | **Participants:** males, 18 years of age or older, meeting criteria for schizophrenia, hospitalised at least once previously for treatment for schizophrenia, currently treated as inpatients on the acute psychiatric ward of a psychiatric hospital.  **Exclusion Criteria:** females. | At least one incident of physical violence against others, objects or the self, or, use of verbal threats preceding admission or present during first presentation. | DSM-IV | None Stated | Schizophrenia | 40 | 70 |
| **Kotler, 1999** [47] | Israel | **Participants:** males and females, 18 years of age or older, meeting criteria for schizophrenia, treated as an inpatient in a general psychiatric hospital (non-violent group) or in a maximum-security forensic psychiatric hospital (violent group).  **Exclusion Criteria:** none stated. | Committed homicide. | ICD-10 | None Stated | Schizophrenia | 30 | 92 |
| **Krakowski, 1989** [48] | USA | **Participants:** males and females, 18 years of age or older, meeting criteria for schizophrenia, treated as inpatients in a regular ward of a psychiatric hospital (non-violent group) or a ward specially designed for the management of violent patients (violent group).  **Exclusion Criteria:** individuals who were too ill or too violent to comply with the study procedure, and those who refused consent. | Two or more incidents of assault against objects or others or verbal threats to kill others during the month prior to study enrolment which were judged to be so serious they necessitated transfer to a special ward designed for the management of violent patients. | DSM-III | None Stated | Schizophrenia | 28^[[14]](#endnote-14)^ | 62 |
| **Krakowski, 1999** [49] | USA | **Participants:** males and females, between 18 and 55 years of age, meeting criteria for schizophrenia or schizoaffective disorder, inpatients in one of two psychiatric hospitals.  **Exclusion Criteria:** individuals diagnosed with systemic (e.g. pulmonary or cardiovascular) diseases. | An incident of physical assault including in the first two months of admission followed by another physical assault during a four week survey period. Assaults included: striking, kicking, pushing or scratching another. | DSM-III-R | SCID | Schizophrenia or  schizoaffective disorder | 44^[[15]](#endnote-15)^ | 125 |
| **Krakowski, 2004a** [50] | USA | **Participants:** males and females, between 18 and 55 years of age, meeting criteria for schizophrenia, schizoaffective or bipolar disorder, treated as inpatients in one of two psychiatric hospitals.  **Exclusion Criteria:** individuals diagnosed with neurological illness or mental retardation. | At least one incident of physical assault including in the first two months of admission Assaults included: striking, kicking, pushing or scratching another. | DSM-III-R | SCID | Schizophrenia, schizoaffective disorder or bipolar disorder | 189 | 1,487 |
| **Krakowski, 2004b** [51] | USA | **Participants:** males and females, between 18 and 55 years of age, meeting criteria for schizophrenia, schizoaffective or bipolar disorder, treated as inpatients in one of two psychiatric hospitals.  **Exclusion Criteria:** individuals who refused consent to participate. | At least one incident of physical assault including in the first two months of admission. Assaults included: striking, kicking, pushing or scratching another. | DSM-III-R | SCID | Schizophrenia, schizoaffective disorder or bipolar disorder | 216 | 297^[[16]](#endnote-16)^ |
| **Krakowski, 2006** [52] | USA | **Participants:** males and females, between 18 and 60 years of age, meeting criteria for schizophrenia or schizoaffective disorder, treated as an inpatient in a psychiatric hospital, at least one physical assault against another during the current inpatient admission in addition to a history of physical assault prior to study enrolment, participating in a randomised controlled trial of clozapine, olanzapine or haloperidol for the management of acute inpatient aggression.  **Exclusion Criteria:** individuals hospitalised for more than one year, those with a history of non-response to clozapine, olanzapine or haloperidol, patients with a history of clozapine, olanzapine, or haloperidol intolerance, those currently receiving contraindicated medications, and those who had received depot antipsychotics within 30 days prior to study enrolment. | Scores on the physical aggression sub-scale of the MOAS. | DSM-IV | None Stated | Schizophrenia & schizoaffective disorder |  | 110^[[17]](#endnote-17)^ |
| **Kumari, 2006** [53] | UK | **Participants:** males, between 18 and 55 years of age, meeting criteria for schizophrenia, treated as inpatients in psychiatric hospitals or in outpatient clinics (non-violent group) or incarcerated in a forensic psychiatric hospital or unit (violent group).  **Exclusion Criteria:** females, left handers, individuals who abused substances prior to study recruitment, or who were diagnosed with comorbid antisocial personality disorder, or those with a history of neurological conditions or head injury, or those whose first language is a language other than English. | The most recent incident of violence (index offence) scored four or more on the Gunn and Robertson Scale for Violence, indicative of a fatal or near-fatal attack on the victim. | DSM-IV | SCID | Schizophrenia | 12 | 25 |
| **Kumari, 2009a** [54] | UK | **Participants:** males, between 18 and 55 years of age, meeting criteria for schizophrenia, treated as inpatients in psychiatric hospitals or in outpatient clinics (non-violent group) or incarcerated in a forensic psychiatric hospital or unit (violent group).  **Exclusion Criteria:** females, left handers, individuals who abused substances prior to study recruitment, or were diagnosed with comorbid antisocial personality disorder, or with a history of neurological conditions or head injury, and those whose first language is a language other than English. | The most recent incident of violence (index offence) scored five or more on the Gunn and Robertson Scale for Violence, indicating a fatal or near-fatal attack on the victim. | DSM | SCID | Schizophrenia | 13 | 26 |
| **Kumari, 2009b** [55] | UK | **Participants:** males, between 18 and 55 years of age, meeting criteria for schizophrenia, treated as inpatients in psychiatric hospitals or at outpatient clinics (non-violent group) or incarcerated in a forensic psychiatric hospital or unit (violent group).  **Exclusion Criteria:** females, left handers, individuals who abused substances prior to study recruitment, or were diagnosed with comorbid antisocial personality disorder, or with a history of neurological conditions or head injury, and those whose first language is a language other than English. | The most recent incident of violence (index offence) scored five or more on the Gunn and Robertson Scale for Violence, indicating a fatal or near-fatal attack on the victim. | DSM | SCID | Schizophrenia | 10 | 24 |
| **Lachman, 1998** [56] | USA | **Participants:** males and females, 18 years of age or older, meeting criteria for schizophrenia or schizoaffective disorder, the majority of whom were treated as inpatients in one of two psychiatric centres.  **Exclusion Criteria:** individuals with only a single assault, those who use aggressive gestures only, or, those who use verbally threatening behaviour only. | History of committing multiple physical assaults against others, including: hitting, kicking, slapping, biting, choking, use of a weapon or engaging in threatening behaviour. | DSM-IV | None Stated | Schizophrenia or schizoaffective disorder | 27 | 55 |
| **Lafayette, 2003** [57] | USA | **Participants:** males and females, 18 years of age or older, meeting criteria for schizophrenia or schizoaffective disorder, treated as outpatients at a mental health clinic between 1999 and 2001.  **Exclusion Criteria:** none stated. | History of at least one arrest for any of the following offences: murder, rape, robbery or aggravated assault. | DSM-IV | SCID | Schizophrenia or schizoaffective disorder | 34 | 57 |
| **Lincoln, 2008** [58] | IC | **Participants:** males and females, 18 years of age or older, meeting criteria for schizophrenia, recently discharged from one of four general (non-violent group) or forensic (violent group) psychiatric hospitals and followed in the community for up to two years post-discharge.  **Exclusion Criteria:** none stated. | At least one incident of the following behaviours committed during the two year follow-up period: throwing objects at others, pushing, shoving, grabbing, slapping, kicking, biting, choking or hitting another, rape, and using a weapon in a threatening manner. | DSM-IV | SCID | Schizophrenia | 27 | 216 |
| **Lysaker, 2002** [59] | USA | **Participants:** males, 18 years of age or older, meeting criteria for schizophrenia or schizoaffective disorder, treated as outpatients, in a stable phase of their illness,.  **Exclusion Criteria:** females, individuals diagnosed with an organic brain syndrome of with a history of mental retardation. | Average of the scores on the assault and verbal sub-scales of the Buss-Durkee Hostility Inventory, indicative of high levels of hostile behaviour expressed directly either through violent actions or through violent verbal threats. | DSM-IV | SCID | Schizophrenia or schizoaffective disorder |  | 36^xiii^ |
| **Maguire, 1997** [60] | Australia | **Participants:** males and females, 18 years of age or older, meeting criteria for schizophrenia, treated as an inpatient within a rehabilitation ward.  **Exclusion Criteria:** individuals not currently stabilised on medication, not currently experiencing at least one positive symptom, with a current diagnosis of substance abuse, a history of head injury or intellectual impairment, and those unable to communicate effectively in English. | Two or more incidents of physical aggression towards objects or persons during a ward survey conducted prior to study enrolment, and at least one incident of physical aggression towards objects or persons each month between the conclusion of this ward survey and the commencement of the study. | DSM-III-R | SCID | Schizophrenia | 20 | 40 |
| **Majorek, 2009** [61] | Germany | **Participants:** males and females, 18 years of age or older, meeting criteria for schizophrenia, treated as inpatients in regular psychiatric hospitals (non-violent group) or incarcerated in a high-security forensic psychiatric hospital (violent group).  **Exclusion Criteria:** none stated. | Convicted or awaiting trial for offences including: theft/robbery, arson, unlawful coercion, assault, sexual offences, causing severe bodily harm and manslaughter. | DSM-IV | Not Stated | Schizophrenia | 33 | 71 |
| **Martinez-Martin, 2011** [62] | Spain | **Participants:** males and females, 18 years of age or older, meeting criteria for schizophrenia, treated as outpatients in any publically funded mental health centre between April to December, 2005, in a stable phase of their illness, and prescribed at least one second-generation antipsychotic medication for a minimum of three months prior to study enrolment.  **Exclusion Criteria:** individuals without a medical history at the clinic and those who refused or withdrew consent. | An incident of aggression in the week prior to study enrolment which scored at least three on all four sub-scales of the MOAS. | DSM-IV-TR | Not Stated | Schizophrenia | 41 | 895 |
| **Milton, 2001** [63] | UK | **Participants:** males and females, between 16 and 64 years of age, meeting criteria for a psychotic illness, making first contact with a community-based clinic between June 1992 and May 1994 and followed-up for up to three years post-presentation.  **Exclusion criteria:** individuals diagnosed with organic psychosis, and those who withdrew consent. | An incident of aggression including use of a weapon, threatening another with a weapon in hand, sexual assault, and any assault which caused harm to the victim at any point post-presentation. | ICD-10 | RDC | First episode psychosis | 35^[[18]](#endnote-18)^ | 166 |
| **Mitchell, 2011** [64] | UK | **Participants:** males and females, between 18 and 64 year of age, meeting criteria for schizophrenia, schizoaffective disorder, psychosis NOS or delusional disorder, treated as outpatients by a community mental health team (non-violent group) or a forensic community mental health team, or, incarcerated in a medium- or low-secure forensic psychiatric hospital (violent group).  **Exclusion Criteria:** individuals who were acutely psychotic at the time of the study, those diagnosed with an autistic-spectrum disorder or another learning disability, those with a history of organic or acquired brain injury and those whose first language is a language other than English. | History of violence against others which would present a significant risk of injury to the victim. | Not Stated | Not Stated | Schizophrenia, schizoaffective disorder, psychosis NOS or delusional disorder | 18 | 29 |
| **Modai, 2000** [65] | Israel | **Participants:** males and females, 18 years of age and older, meeting criteria for schizophrenia, treated as inpatients.  **Exclusion Criteria:** individuals who had been prescribed antidepressants, atypical antipsychotics, lithium or aspirin within the three months prior to study enrolment and those diagnosed with a major physical illness, drug dependency, epilepsy or organic brain disorders. | At least one incident of aggressive behaviour in the six months preceding study enrolment. | DSM-IV | Not Stated | Schizophrenia | 11^[[19]](#endnote-19)^ | 26 |
| **Monahan, 2000** [66] | USA | **Participants:** males and females, between 18 and 40 years of age, meeting criteria for schizophrenia, recently released from in one of three acute inpatient psychiatric hospitals, and followed for up to 20 weeks in the community.  **Exclusion Criteria:** individuals unable to communicate effectively in English, and those of an ethnicity other than white, African American or Hispanic. | At least one incident of battery, sexual assault, use of a weapon, and threats made with a weapon in the ten weeks prior to discharge. | DSM-III-R | Not Stated | Schizophrenia^[[20]](#endnote-20)^ | 13 | 160 |
| **Munkner, 2005** [67] | Denmark | **Participants:** males and females, 15 years of age or older, born on or after 1 November, 1963, meeting criteria for schizophrenia, treated as an inpatient or outpatient, followed in the community for up to twenty years post-treatment.  **Exclusion Criteria:** individuals who died during the follow-up period and those admitted to a psychiatric hospital before the age of 15. | Convicted for a violent offence, including: homicide, attempted homicide, aggravated assault, common assault, illegal threats, arson, rape, attempted rape, other sexual offences, robbery, and any other violent interpersonal offence. This conviction must have occurred after diagnosis for schizophrenia. | ICD-8  or -10 | None Stated | Schizophrenia | 215 | 4,035^[[21]](#endnote-21)^ |
| **Nederlof, 2011** [68] | Netherlands | **Participants:** males and females, between 19 and 51 years of age, meeting criteria for schizophrenia, schizoaffective disorder, delusional disorder or psychosis NOS, treated as inpatients in one of three psychiatric hospitals between April, 2008 and December, 2009.  **Exclusion criteria:** individuals diagnosed with a comorbid axis I or II disorder, currently suffering from cognitive distortions or receiving tranquilising medications capable of distorting alertness and attention. | Total score on the Aggression Questionnaire. | DSM-IV | SCID-I/P | Schizophrenia, schizoaffective disorder, delusional disorder or psychosis NOS |  | 124^xiii^ |
| **Nolan, 1999** [69] | USA | **Participants:** males and females, 18 years of age or older, Caucasian, meeting criteria for schizophrenia or schizoaffective disorder, treated as inpatients at one of two psychiatric centres.  **Exclusion criteria:** individuals of an ethnicity other than Caucasian, those who were violent whilst acutely psychotic or who had been not violent during the one year preceding study enrolment. | At least two assaults against others as documented in medical records, admissions or discharge summaries or RAP sheets. | DSM-IV | None Stated | Schizophrenia or Schizoaffective Disorder | 26 | 51 |
| **Nolan, 2000** [70] | USA | **Participants:** males and females, 18 years of age or older, Caucasian, meeting criteria for schizophrenia or schizoaffective disorder, treated as inpatients at one of four psychiatric centres.  **Exclusion criteria:** individuals of an ethnicity other than Caucasian, those who were violent whilst acutely psychotic or who had been not violent during the one year preceding study enrolment, and those without at least one psychiatric inpatient admission preceding study enrolment. | At least two assaults against others as documented in medical records, admissions or discharge summaries or RAP sheets. | DSM-IV | None Stated | Schizophrenia or  Schizoaffective Disorder | 42 | 84 |
| **Nolan, 2005** [71] | USA | **Participants:** males and females, between 18 and 60 years of age, meeting criteria for schizophrenia or schizoaffective disorder, inpatients at one of four state psychiatric facilities, have a minimum score of 60 on the PANSS at baseline, have persistent positive symptoms of psychosis at baseline which have continued unabated despite six consecutive weeks of treatment with at least one conventional antipsychotic medication at 600mg chlorpromazine equivalent dosage levels, have poor functional recovery as indicated by a lack of employment, education or significant relationships outside the family in the two years prior to study enrolment. Participants were participating in a 14-week randomised controlled trial of clozapine, olanzapine, risperidone or haloperidol.  **Exclusion criteria:** none stated. | At least one incident of aggression during the 14 week study period as recorded on the OAS. | DSM-IV | None Stated | Schizophrenia, or schizoaffective disorder | 88 | 157 |
| **Oldemeyer, 2009** [72] | USA | **Participants:** males, 18 years of age and older, meeting criteria for schizophrenia, schizoaffective disorder, psychosis NOS or bipolar disorder, incarcerated for a non-violent offence (non-violent group) or for a violent offence (violent group).  **Exclusion Criteria:** females. | Conviction for arson, assault, kidnapping, rape, other sexual assaults or murder. | DSM-IV-TR | None Stated | Schizophrenia, schizoaffective disorder, psychosis NOS or bipolar disorder | 29 | 72 |
| **Ran, 2010** [73] | China | **Participants:** males and females, 15 years of age and older, meeting criteria for schizophrenia, followed in the community for up to 10 years after study enrolment.  **Exclusion Criteria:** none stated. | At least one incident of violent property damage, arson, sexual assaults, physical assaults or murder during the 10 year follow-up period. | ICD-10 | PSE-9 | Schizophrenia | 49 | 489 |
| **Rasanen, 1998** [74] | Finland | **Participants:** males, born in 1966, living in Finland until at least 16 years of age, meeting criteria for schizophrenia, schizophreniform disorder, schizoaffective disorder, delusional disorder, schizoid personality disorder or schizotypal personality disorder.  **Exclusion Criteria:** females, members of the 1966 unselected birth cohort who did not live in Finland until at least 16 years of age. | Conviction for homicide, assault, robbery, arson or violation of the peace before at least 27 years of age. | DSM-III-R | OPCRIT | Schizophrenia, schizophreniform disorder, schizoaffective disorder, delusional disorder, schizoid personality disorder or schizotypal personality disorder. | 11 | 86 |
| **Rasmussen, 1995** [75] | Norway | **Participants:** males, between 20 and 45 years of age, meeting criteria for schizophrenia, treated as inpatients or outpatients of a general psychiatric hospital (non-violent group), or, treated as inpatients in a special psychiatric unit for violent and aggressive patients (violent group).  **Exclusion Criteria:** individuals who were too ill to comply with the testing procedure. | At least one prior incident of violence or aggression which necessitated admission to a special unit for the management of violent or aggressive patients. | DSM-III | None Stated | Schizophrenia | 13 | 26 |
| **Ritsner, 2003** [76] | Israel | **Participants:** males and females, between 20 and 57 years of age, meeting criteria for schizophrenia, treated as inpatients in a general psychiatric institution.  **Exclusion Criteria:** individuals prescribed antidepressants, mood stabilisers or benzodiazepines within three months prior to study enrolment, participants suffering from any major physical illness, participants with a history of drug or alcohol abuse, a history of epilepsy, or, a history of organic brain syndromes. | At least four incidents of physical aggression over the two months prior to study enrolment. | DSM-IV | SCID | Schizophrenia | 11^[[22]](#endnote-22)^ | 25 |
| **Roy, 1987** [77] | USA | **Participants:** males, between 25 and 44 years of age, meeting criteria for schizophrenia, treated as inpatients, previously resistant to antipsychotic treatment with at least three different antipsychotics (at least one being a typical antipsychotic and at least one being an atypical antipsychotic) as defined by a failure to demonstrate an improvement in symptoms for a minimum of six weeks over a period of two and a half years prior to study enrolment, currently prescribed a fixed dose of a high-potency antipsychotic medication (haloperidol) three times daily.  **Exclusion Criteria:** females. | Score of three or greater on LSIV. | DSM-III | None Stated | Schizophrenia | 11 | 20 |
| **Sarne, 1995** [78] | Israel | **Participants:** males and females, 18 years of age or older, meeting criteria for schizophrenia, treated as inpatients.  **Exclusion Criteria:** individuals prescribed antidepressents, lithium or benzodiazepines. | At least one incident of hostile behaviour both during the current admission and during previous inpatient admissions as recorded in the participants’ medical records. | DSM-III | None Stated | Schizophrenia | 12 | 24 |
| **Schanda, 1992** [79] | Austria | **Participants:** males, 18 years of age or older, meeting criteria for schizophrenia, inpatients in an urban or rural general psychiatric hospital (non-violent group) or incarcerated in a secure hospital for mentally ill offenders (violent group).  **Exclusion Criteria:** females. | Participants convicted for murder, attempted murder, severe bodily injury, compulsion, illegal threats, robbery, sexual delinquency and arson. | ICD-9 | None Stated | Schizophrenia | 28 | 51 |
| **Schug, 2010** [80] | China | **Participants:** males and females, 18 years of age and older, meeting criteria for schizophrenia, acute transient psychosis with paranoia, acute transient psychosis with a schizophrenia-like psychosis or schizophrenia-like psychosis, treated as inpatients in a general hospital (non-violent group) or detained in a forensic psychiatric unit (violent group).  **Exclusion Criteria:** none stated. | Accused of homicide. | DSM-IV | None Stated | Various schizophrenia-spectrum diagnoses. | 32 | 65 |
| **Serper, 2008** [81] | USA | **Participants:** males and females, between 18 and 65 years of age, meeting criteria for schizophrenia, schizoaffective disorder or bipolar disorder, treated as an inpatient in one of six acute inpatient psychiatric units within 72 hours of admission due to an acute exacerbation of illness.  **Exclusion Criteria:** individuals diagnosed with mental retardation, dementia, HIV, any disorder of the central nervous system, a developmental disorder, or any other disorder which may affect cognitive functioning, and those with a history of head trauma, or whose level of reading was below the sixth-grade. | Scores on the Retrospective Overt Aggression Scale (ROAS). | DSM-IV | SCID | Schizophrenia, schizoaffective disorder or bipolar disorder |  | 85^xiii^ |
| **Silver, 2005** [82] | Israel | **Participants:** males, 18 years of age or older, meeting criteria for schizophrenia or schizoaffective disorder, treated as inpatients in a regular ward of a general psychiatric hospital (non-violent group) or in a maximum security ward of the same hospital which specialises in the treatment of violent offenders who continue to regularly behave violently in standard psychiatric institutions (violent group) for at least six months prior to study enrolment.  **Exclusion Criteria:** individuals with a duration of illness for less than two years, comorbid major depression, a history of brain damage, and those who abused drugs or alcohol during the six months prior to study enrolment. | Convicted of murder, rape or recurrent acts of violence against others (e.g. robbery, assaults) and behaviour during admission which was so violent it necessitated transfer to a maximum security ward specialising in the treatment of violent patients. | DSM-IV | None Stated | Schizophrenia or  schizoaffective disorder | 35 | 70 |
| **Song, 2009** [83] | South Korea | **Participants:** males and females, between 18 and 60 years of age, meeting criteria for schizophrenia, schizoaffective disorder or schizophreniform disorder, treated as outpatients or inpatients at a general medical centre.  **Exclusion criteria:** individuals younger than 18 or older than 60 years of age, those diagnosed with an axis I psychiatric disorder other than schizophrenia, have an intelligence quotient of less than 70, or those unable to complete the test battery for other reasons. | Scores on the MOAS. | DSM-IV | SCID | Schizophrenia, schizoaffective disorder, or schizophreniform disorder |  | 105^xiii^ |
| **Soyka, 2007** [84] | Germany | Participants: males and females, 18 years of age or older, meeting criteria for schizophrenia, treated as inpatients between 1990 and 1995 at a University psychiatric hospital and now living in the community.  **Exclusion Criteria:** none stated. | Convicted of a violent offence, including: assault, aggravated assault, aggravated battery, illegal threats, invasion, sexual abuse of a minor, robbery, attempted manslaughter, illegal restraint, involuntary manslaughter, manslaughter, murder, sexual assault, rape, use of a weapon between discharge from inpatient care and 2002. | ICD-9 | None Stated | Schizophrenia | 62 | 1,555 |
| **Spidel, 2010** [85] | Canada | **Participants:** males and females, 18 years of age or older, meeting criteria for a schizophrenia-spectrum psychosis or with bipolar disorder, treated as an outpatient by one of two first-episode psychosis services, resident in the community, with an onset of psychosis symptoms for less than two years prior to study enrolment.  **Exclusion criteria:** individuals with an intellectual disability and those diagnosed with an organic disorder or drug induced psychosis. | A lifetime history of physical aggression as recorded on the physical aggression subscale of the MOAS. | DSM-IV | None Stated | Schizophrenia, Schizoaffective disorder, bipolar disorder, or psychosis NOS | 50^[[23]](#endnote-23)^ | 118 |
| **Steinert, 1996** [86] | Germany | **Participants:** males and females, 18 years of age or older, meeting criteria for schizophrenia, treated as inpatients a state psychiatric hospital.  **Exclusion Criteria:** individuals with a history of aggressive behaviour during previous psychiatric inpatient treatment (non-violent group only). | Participants who were responsible for at least one incident of aggression during treatment in the acute psychiatric reception ward as recorded on the OAS. | ICD-10 | None Stated | Schizophrenia | 53 | 83^[[24]](#endnote-24)^ |
| **Steinert, 1999** [87] | Germany | **Participants:** males and females, 18 years of age or older, meeting criteria for a first-episode of schizophrenia or schizoaffective disorder, treated as inpatients in a University psychiatric hospital between 1990 and 1993.  **Exclusion criteria:** individuals whose diagnosis was unclear, who lived outside catchment area, who had experienced less than one month of continuous psychotic symptomatology prior to study enrolment, participated less than 14 years of age or over 60 years of age, and those diagnosed with a progressive organic brain disease. | Scores of at least one on three of the four MOAS subscales; the self-directed aggression subscale was not considered. | ICD-10 | None Stated | Schizophrenia or Schizoaffective Disorder | 90 | 138 |
| **Stompe, 2004** [88] | Austria | **Participants:** males, 18 years of age or older, meeting criteria for schizophrenia, treated as inpatients in a University psychiatric clinic (non-violent group) or incarcerated in a high-security forensic psychiatric facility (violent group).  **Exclusion Criteria:** females, members of the non-violent group who had a conviction for a violent offence or whose medical records indicated incidents of aggression or violence prior to study enrolment. | Found not guilty by reason of insanity for any violent offence, including: sexual offences, robbery, compulsion using a weapon, severe property damage, causing severe bodily harm, arson, murder and attempted murder. | DSM-IV | None Stated | Schizophrenia | 119 | 224 |
| **Stompe, 2006** [89] | Austria | **Participants:** males, between 18 and 62 years of age, meeting criteria for schizophrenia, treated as inpatients in a University psychiatric clinic or rehabilitation clinic (non-violent group) or incarcerated in a high-security forensic psychiatric facility (violent group).  **Exclusion Criteria:** females, members of the non-violent group who had a conviction for a violent offence or whose medical records indicated incidents of aggression or violence prior to study enrolment. | Found not guilty by reason of insanity for any violent offence, including: sexual offences, robbery, compulsion using a weapon, severe property damage, causing severe bodily harm, arson, murder and attempted murder. | DSM-III | SCID-I | Schizophrenia | 103 | 206 |
| **Swanson, 2000** [90] | USA | **Participants:** males and females, 18 years of age and older, meeting criteria for a major mental illness ( 96% met criteria for schizophrenia, schizoaffective disorder, another psychotic disorder or bipolar disorder), a duration of illness of at least one year preceding study enrolment, experience significant functional disruption to daily living, receiving intensive treatment for psychosis at any point during the two years preceding study enrolment, participating in a randomised controlled trial of involuntary outpatient commitment, followed for up to one year in the community.  **Exclusion Criteria:** none stated. | At least one incident of being picked up by police or arrested for assault, fights involving physical contact or threats with a weapon in hand as reported by either the self or a collateral informant during the one year follow-up period. | DSM | None Stated | 96% of the sample have a diagnosis of a psychotic spectrum disorder, including: Schizophrenia, schizoaffective disorder, another psychosis-spectrum disorder or bipolar disorder | 100 | 262 |
| **Swanson, 2002** [91] | USA | **Participants:** males and females, 18 years of age or older, meeting criteria for schizophrenia or schizoaffective disorder, treated as outpatients or inpatients at public mental health facilities in one of four states.  **Exclusion Criteria:** none stated. | At least one incident of fighting, assault causing bodily injury to the victim, use of a weapon to threaten or harm the victim and any sexual assault during the one year preceding study enrolment. | DSM-IV | None Stated | Schizophrenia or schizoaffective disorder^[[25]](#endnote-25)^ | 114 | 514 |
| **Swanson, 2004** [92] | USA | **Participants:** males and females, 18 years of age or older, meeting criteria for schizophrenia, schizoaffective disorder or schizophreniform disorder, treated in the community between 1999 and 2001.  **Exclusion Criteria:** individuals who were unable to provide informed consent or had participated in a clinical trial within 30 days prior to study enrolment. | An incident of assault or battery (e.g. hitting, shoving, kicking or biting) against another as reported by either the self, or, as recorded in medical records or arrest records. | DSM-IV | None Stated | Schizophrenia, schizoaffective disorder, or schizophreniform disorder^xxxv^ | 67 | 402 |
| **Swanson, 2006** [93] | USA | **Participants:** males and females, between 18 and 65 years of age, meeting criteria for any major psychosis-spectrum disorder, treated as outpatients by any publically funded mental health service in one of five cities, with an onset of psychosis at least six months prior to study enrolment.  **Exclusion criteria:** individuals treated for substance use disorders only, and those unable to speak either English or Spanish. | At least one incident of violence, including: assault (regardless of whether the assault caused injury to the victim), use of a weapon, use of a weapon which caused injury to the victim, threats with a weapon in hand and sexual assault. | DSM-IV | None Stated | Various psychosis-spectrum disorders^xxxv^ | 59 | 1011 |
| **Swanson, 2006** [94] | USA | **Participants:** males and females, between 18 and 65 years of age, meeting criteria for schizophrenia, treated as outpatients or inpatients in one of 57 clinical sites located throughout the country between 2001 and 2004, symptomatically stable enough to be treated with oral medications only.  **Exclusion Criteria:** individuals meeting criteria for first episode psychosis, schizoaffective disorder, mental retardation, developmental disorders or neurodegenerative disorders, participants with other medical conditions particularly cardiac conditions, participants with a history of serious adverse reactions to antipsychotics, participants with a history of failing to respond to antipsychotic medications, those with treatment resistant psychosis, those stabilised on depot antipsychotic medications, women who are pregnant or breast-feeding, individuals with tardive dyskinesia and those with a contraindication to any antipsychotic medication. | At least one incident of violence, including: assault (regardless of the level of injury caused to the victim), use of a weapon, use of a weapon which caused injury to the victim, threats with a weapon in hand and sexual assault. | DSM-IV | SCID | Schizophrenia^xxxv^ | 270 | 1,410 |
| **Swanson, 2008** [95] | USA | **Participants:** males and females, between 18 and 65 years of age, meeting criteria for schizophrenia, treated as outpatients or inpatients in one of 57 clinical sites located throughout the country between 2001 and 2004, symptomatically stable enough to be treated with oral medications only, received a minimum of six months treatment.  **Exclusion Criteria:** individuals meeting criteria for first episode psychosis, schizoaffective disorder, mental retardation, developmental disorders or neurodegenerative disorders, participants with other medical conditions particularly cardiac conditions, participants with a history of serious adverse reactions to antipsychotics, participants with a history of failing to respond to antipsychotic medications, those with treatment resistant psychosis, those stabilised on depot antipsychotic medications, women who are pregnant or breast-feeding, individuals with tardive dyskinesia and those with a contraindication to any antipsychotic medication. | At least one incident of violence, including: assault (regardless of the level of injury caused to the victim), use of a weapon, use of a weapon which caused injury to the victim, threats with a weapon in hand and sexual assault. | DSM-IV | SCID-I/P | Schizophrenia | 202 | 1,445 |
| **Teixeria, 2009** [96] | Brazil | **Participants:** males, 18 years of age or older, meeting criteria for a schizophrenia-spectrum disorder with prominent delusional ideas for a minimum of five years but less than 20 years, treated as inpatients in one of two general psychiatric wards in University psychiatric hospitals or in a general psychiatric hospital (non-violent group), or, incarcerated in a high security forensic psychiatric hospital (violent group).  **Exclusion Criteria:** females, individuals whose delusional ideas resolve with treatment with medications, who were diagnosed with substance-induced psychosis or brief psychotic episodes, those who had been diagnosed with a schizophrenia-spectrum disorder for less than five years or greater than 20 years, those with a history of head injury, brain damage or mental retardation, individuals whose violent crime was not directly motivated by their delusional ideas (violent group only), and those who had committed violent offences in the past (non-violent group only). | Found not guilty by reason of insanity of a violent offence including: offences which resulted in the death of the victim (e.g. murder, manslaughter), offences which caused severe harm to the victim (e.g. rape), or, offences of repeated threat of harm to the victim (e.g. muggings). | DSM-IV-TR or ICD-10 | None Stated | Schizophrenia or delusional disorder | 30 | 60 |
| **Thomas, 2005** [97] | UK | **Participants:** males and females, between 18 and 65 years of age, hospitalised on at least two separate occasions for psychotic symptoms, meeting criteria for schizoaffective disorder, schizophrenia, psychosis not otherwise specified or bipolar disorder, participating in a randomised controlled trial of intensive community case management at one of four urban mental health services, and followed for two years whilst living in the community.  **Exclusion Criteria:** individuals diagnosed with an organic brain disorder, or with a history of substance misuse. | An incident of actual physical assault over the two year follow-up period. | ICD-10 | OPCRIT | Schizophrenia, schizoaffective disorder, affective psychosis and unspecified functional psychosis | 158 | 708 |
| **Tripathi, 2010** [98] | India | **Participants:** males, between 18 and 50 years of age, meeting criteria for schizophrenia, treated as inpatients in a general psychiatric facility (non-violent group) or incarcerated in jail in one of two states (violent group).  **Exclusion Criteria:** individuals with a diagnosis other than schizophrenia, with organic pathology, a history of head injury or a history of seizures, those diagnosed with mental retardation, substance abuse or formal thought disorder, individuals unable to communicate effective due to, for example, a tendency towards incoherent speech, and those with less than a seventh grade education. | Convicted of murder, attempted murder, assault, rape, or theft. | ICD-10 | RDC | Schizophrenia | 30 | 60 |
| **Tuninger, 2001** [99] | Sweden | **Participants:** males and females, between 18 and 75 years of age, meeting criteria for a major mental illness (98% met criteria for a psychosis-spectrum disorder), treated as inpatients in a psychiatric ward during the study period of 14 months.  **Exclusion Criteria:** none stated. | Record of a violent offence as recorded in a Government register of criminality. | ICD-9 | SCID | 98% of the sample were diagnosed with a psychosis-spectrum disorder | Unclear | 257 |
| **Turkoglu, 2009** [100] | Turkey | **Participants:** males, between 19 and 59 years of age, meeting criteria for schizophrenia, treated as inpatients at a general psychiatric hospital.  **Exclusion Criteria:** females. | Convicted of violent offences including: homicide, wounding, property damage, assault, battery and robbery. | DSM-IV | None Stated | Schizophrenia | 50 | 100 |
| **Valevski, 1999** [101] | Israel | **Participants:** males and females, 18 years of age or older, meeting criteria for schizophrenia, treated inpatients in a general psychiatric facility (non-violent group) or incarcerated in a high-security forensic psychiatric facility for severely violent psychiatric patients (violent group).  **Exclusion Criteria:** none stated. | Found not guilty by reason of insanity for homicide. | DSM-IV | None Stated | Schizophrenia | 33 | 61 |
| **Verma, 2005** [102] | Singapore | **Participants:** males and females, between 15 and 40 years of age, meeting criteria for a first-episode psychosis, currently experiencing acute psychotic symptoms, treated as an outpatient or inpatient of a specialist first-episode psychosis service.  **Exclusion Criteria:** individuals diagnosed with major medical or neurological illnesses, those currently misusing substances and those who have received treatment with antipsychotics prior to study enrolment. | At least one incident of aggression prior to study enrolment which would score two or more on the MacArthur Community Violence Instrument indicative of weapon use, threats with a weapon, sexual assault or any other act which caused injury to the victim. | DSM-IV | SCID-I | 95% of the sample were diagnosed with schizophrenia, brief psychotic disorder, delusional disorder or psychosis NOS. | 20^[[26]](#endnote-26)^ | 146 |
| **Vevera, 2005** [103] | Czech Republic | **Participants:** males and females, 18 years of age or older, meeting criteria for schizophrenia, treated as inpatients in an inpatient unit in a University psychiatric unit in 1949, 1969, 1989 or 2000.  **Exclusion Criteria:** individuals diagnosed with a mental illness other than schizophrenia, and those from a non-white ethnic background. | At least one incident of physical or verbal aggression which scored three or greater on the MOAS indicative of physically aggression against another person or verbal aggression accompanied by threats with a weapon in hand during the study period. | DSM-IV | None Stated | Schizophrenia | 154 | 404 |
| **Volavka, 1997** [104] | IC | **Participants:** males and females, between 15 and 54 years of age, meeting criteria for schizophrenia, suffering from psychosis symptoms for at least 12 months prior to study enrolment, making first contact with a psychiatric “helping agency” in one of ten countries within three months prior to study enrolment.  **Exclusion Criteria:** individuals who have resided in the catchment area of their local psychiatric “helping agency” for less than six months prior to study enrolment, those who have not suffered from psychosis symptoms, those with a diagnosis other than for schizophrenia, and those with incomplete records on history of assault. | Answering affirmatively to the Psychiatric and Personal History Schedule (PPHS) item: “Did you at any point in the past assault another person physically?” | ICD-10 | None Stated | Schizophrenia | 209 | 1,017 |
| **Wootton, 2008** [105] | UK | **Participants:** males and females, between 18 and 65 years of age, hospitalised on at least two separate occasions for psychotic symptoms, meeting criteria for schizoaffective disorder, schizophrenia, psychosis not otherwise specified or bipolar disorder, participating in a randomised controlled trial of intensive community case management at one of four urban mental health services, and followed for two years whilst living in the community.  **Exclusion Criteria:** males, individuals diagnosed with an organic brain disorder, or those with a history of substance misuse. | An incident of actual physical assault over the two year follow-up period as revealed by self-report, review of case-notes, from case managers’ reports or from criminal records. | ICD-10 | RDC | Schizophrenia, schizoaffective disorder, affective psychosis or unspecified functional psychosis | 158 | 708 |
| **Yang, 2010** [106] | China | **Participants:** males and females, 18 years of age or older, meeting criteria for schizophrenia, treated as inpatients in a general hospital (non-violent group) or incarcerated in a forensic psychiatric unit (violent group).  **Exclusion Criteria:** none stated. | Accused of homicide. | DSM-IV | None Stated | Schizophrenia | 22 | 41 |
| **Yen, 2002** [107] | Taiwan | **Participants:** males and females, 18 years of age or older, meeting criteria for schizophrenia, treated as outpatients in a medical centre or a general psychiatric hospital, currently in remission or with minimal residual psychosis symptomatology as indicated by a score of 60 or less on the PANSS, followed for up to one year.  **Exclusion Criteria:** individuals with comorbid mental retardation, substance use disorder or organic mental disorder. | Scores on the violence dimension of the Violence and Suicide Assessment Scale (VSAS) over the one year follow-up period. | DSM-IV | None Stated | Schizophrenia |  | 74^xiii^ |
| **Yesavage, 1983** [108] | USA | **Participants:** males, between 19 and 68 years of age, meeting criteria for schizophrenia or schizoaffective disorder, treated as inpatients in a psychiatric intensive care unit during a 16 month study period, admitted for inpatient treatment involuntarily.  **Exclusion criteria:** females. | At least one incident of assault-related behaviour observed for up to seven days post-admission, scored according to a modified version of LSIV. | DSM-III | None Stated | Schizophrenia or schizo-affective disorder |  | 207^xiii^ |
| **Yesavage, 1984** [109] | USA | **Participants:** males, between 19 and 68 years of age, meeting criteria for schizophrenia or schizoaffective disorder, treated as inpatients in a psychiatric intensive care unit during a 16 month study period, admitted for inpatient treatment involuntarily.  **Exclusion criteria:** females. | At least one incident of assault-related behaviour observed for up to seven days post-admission which necessitated either seclusion or use of four-point restraints to protect staff or fellow patients from the participant. Incidents were scored according to a modified version of LSIV. | DSM-III | None Stated | Schizophrenia |  | 70^xiii^ |
| **Zoghes, 1988** [110] | Greece | **Participants:** males, between 18 and 65 years of age, meeting criteria for schizophrenia, treated as inpatients at one of two psychiatric clinics within a public psychiatric hospital (non-violent group) or incarcerated in a “prisoner’s unit” within a public psychiatric hospital (violent group).  **Exclusion Criteria:** females, individuals who are illiterate, foreign born, and those diagnosed with a Central Nervous System disease. | Committed murder or attempted murder. | DSM-III | None Stated | Schizophrenia | 67 | 134 |

1. **Table Notes:**

   Analyses limited to participants who were violent. [↑](#endnote-ref-1)
2. Analyses limited to participants who were not hostile. [↑](#endnote-ref-2)
3. Stands for International Collaboration. [↑](#endnote-ref-3)
4. Analyses limited to participants who behaved aggressively during the 6 month treatment period. [↑](#endnote-ref-4)
5. Analyses limited to participants who were convicted for a violent crime. [↑](#endnote-ref-5)
6. Analyses limited to those participants diagnosed with schizophrenia, rather than another first-episode psychosis (e.g. affective psychosis). [↑](#endnote-ref-6)
7. Analyses limited to the second observation period between 4 – 6 months. [↑](#endnote-ref-7)
8. Analyses limited to participants with a conviction for a violent crime. [↑](#endnote-ref-8)
9. Analyses limited to those participants who engaged in physical violence during the week following first-contact with psychiatric services. [↑](#endnote-ref-9)
10. Analyses limited to those participants who were highly aggressive. [↑](#endnote-ref-10)
11. Analyses limited to participants who behaved aggressively in the second six month period post-discharge. [↑](#endnote-ref-11)
12. Analyses llimited to those participants who were actually assaultive during follow-up. [↑](#endnote-ref-12)
13. Study reported correlations; thus all participants received a score on the violence outcome measure. [↑](#endnote-ref-13)
14. Analyses limited to those participants who were highly violent. [↑](#endnote-ref-14)
15. Analyses limited to those participants who were persistently violent. [↑](#endnote-ref-15)
16. Analyses limited to those participants categorized as violent or non-violent. [↑](#endnote-ref-16)
17. All participants were evaluated for physical aggression. [↑](#endnote-ref-17)
18. Analyses limited to those participants who were aggressive during the follow-up period post-presentation. [↑](#endnote-ref-18)
19. Analyses limited to those participants who were currently aggressive. [↑](#endnote-ref-19)
20. Analyses limited to those participants diagnosed with schizophrenia, rather than another major mental illness. [↑](#endnote-ref-20)
21. Data extracted for the period after diagnosis was made. [↑](#endnote-ref-21)
22. Analyses limited to those participants who were repetitively aggressive at the time of the study. [↑](#endnote-ref-22)
23. Analyses limited to those participants who scored highly on the Modified Overt Aggression Scale physical aggression sub-scale. [↑](#endnote-ref-23)
24. Analyses limited to men and women who were aggressive (violent group) and men and women who had no history of aggressive behaviour ever (non-violent group). [↑](#endnote-ref-24)
25. Dr. Richard van Dorn provided data for those participants diagnosed with a psychosis spectrum disorder only. [↑](#endnote-ref-25)
26. Analyses limited to participants meeting criteria for level two aggression.

    **References:**

    1. Abu-Akel A, Abushua'lea K (2004) 'Theory of mind' in violent and nonviolent patients with paranoid schizophrenia. Schizophr Res 69: 45-53.

    2. Abushua'lea K, Abu-Akel A (2006) Association of psychopathic traits and symptomatology with violence in patients with schizophrenia. Psychiatry Res 143: 205-211.

    3. Arango C, Barba AC, González-Salvador T, Ordóñez AC (1999) Violence in inpatients with schizophrenia: A prospective study Schizophr Bull 25: 493-503.

    4. Arango C, Bombín I, González-Salvador T, García-Cabeza I, Bobes J (2006) Randomised clinical trial comparing oral versus depot formulations of zuclopenthixol in patients with schizophrenia and previous violence. Eur Psychiatry 21: 34-40.

    5. Barkataki I, Kumari V, Das M, Hill M, Morris R, et al. (2005) A neuropsychological investigation into violence and mental illness. Schizophr Res 74: 1-13.

    6. Barkataki I, Kumari V, Das M, Taylor PJ, Sharma T (2006) Volumetric structural brain abnormalities in men with schizophrenia or antisocial personality disorder. Behav Brain Res 169: 239-247.

    7. Bartels SJ, Drake RE, Wallach MA, Freeman DH (1991) Characteristic hostility in schizophrenic outpatients. Schizophr Bull 17: 163-171.

    8. Belfrage H (1998) A ten-year follow-up of criminality in Stockholm mental patients Br J Criminol 58: 145-155.

    9. Bitter I, Czobor P, Dossenbach M, Volavka J (2005) Effectiveness of clozapine, olanzapine, quetiapine, risperidone, and haloperidol monotherapy in reducing hostile and aggressive behavior in outpatients treated for schizophrenia: A prospective naturalistic study (IC-SOHO). Eur Psychiatry 20: 403-408.

    10. Bobes J, Fillat O, Arango C (2009) Violence among schizophrenia out-patients compliant with medication: Prevalence and associated factors. Acta Psychiatr Scand 119: 218-225.

    11. Buckley PF, Hrouda DR, Friedman L, Noffsinger SG, Resnick PJ, et al. (2004) Insight and its relationship to violent behaviour in patients with schizophrenia Am J Psychiatry 161: 1712-1714.

    12. Calcedo-Barba AL, Calcedo-Ordóñez A (1994) Violence and paranoid schizophrenia. Int J Law Psychiatry 17: 253-263.

    13. Cannon M, Huttunen MO, Tanskanen AJ, Arseneault L, Jones PB, et al. (2002) Perinatal and childhood risk factors for later criminality and violence in schizophrenia. Br J Psychiatry 180: 496-501.

    14. Chang WC, Tang JYM, Hui CLM, Chiu CPY, Lam MML, et al. (2011) Gender differences in patients presenting with first episode psychosis in Hong Kong: A three-year follow-up study. Aust N Z J Psychiatry 45: 199-205.

    15. Cheung P, Schweitzer I, Crowley KC, Yastrubetskaya O, Tuckwell V (1996) Aggressive behaviour and extrapyramidal side effects of neuroleptics in schizophrenia. Int Clin Psychopharmacol 11: 237-240.

    16. Cheung P, Schweitzer I, Crowley K, Tuckwell V (1997a) Aggressive behaviour in schizophrenia: The role of psychopathology. Aust N Z J Psychiatry 31: 62-67.

    17. Cheung P, Schweitzer I, Crowley K, Tuckwell V (1997b) Violence in schizophrenia: Role of hallucinations and delusions Schizophr Res 26: 181-190.

    18. Cheung P, Schweitzer I, Crowley K, Tuckwell V (1997c) Aggressive behaviour in schizophrenia: Role of state versus trait factors. Psychiatry Res 72: 41-50.

    19. Chung S, Lee T-K, Jung J, Chang JK, Jang SH, et al. (2006) Clinical characteristics of schizophrenic patients who committed homicide. J Korean Neuropsychiatr Assoc 45: 11-20.

    20. Chung S, Chung HY, Jung J, Chang JK, Hong JP (2010) Association among aggressiveness, neurocognitive function, and the Val66Met polymorphism of brain-derived neurotrophic factor gene in male schizophrenic patients Compr Psychiatry 51: 367-372.

    21. Cuffel BJ, Shumway M, Chouljian TL, MacDonald T (1994) A longitudinal study of substance use and community violence in schizophrenia. J Nerv Ment Dis 182: 704-708.

    22. Dean K, Walsh E, Moran P, Tyrer P, Creed F, et al. (2006) Violence in women with psychosis in the community: Prospective study. Br J Psychiatry 188: 264-270.

    23. Dean K, Walsh E, Morgan C, Demjaha A, Dazzan P, et al. (2007) Aggressive behaviour at first contact with services: Findings from the AESOP First Episode Psychosis Study. Psychol Med 37: 547-557.

    24. Ellouze F, Ayedi S, Masmoudi S, Bakri L, Cherif W, et al. (2009) Schizophrenia and violence: Incidence and risk factors: A Tunisian sample. Encephale 35: 347-352.

    25. Eriksson A, Romelsjö A, Stenbacka M, Tengström A (2010) Early risk factors for criminal offending in schizophrenia: A 35-year longitudinal cohort study. Soc Psychiatry Epidemiol 46: 925-932.

    26. Erkiran M, Özünalan H, Evren C, Aytaçlar S, Kirisci L, et al. (2006) Substance abuse amplifies the risk for violence in schizophrenia spectrum disorder. Addictive Behaviors 31: 1797-1805.

    27. Fazel S, Grann M, Carlström E, Lichtenstein P, Långström N (2009) Risk factors for violent crime in schizophrenia: A national cohort study of 13,806 patients J Clin Psychiatry 70: 362-369.

    28. Fazel S, Buxrud P, Ruchkin V, Grann M (2010) Homicide in discharged patients with schizophrenia and other psychoses: A national case-control study. Schizophr Res 123: 263-269.

    29. Flannery RB, Penk WE, Irvin EA, Gallagher C (1998) Characteristics of violent versus nonviolent patients with schizophrenia. Psychiatr Q 69: 83-93.

    30. Foley SR, Kelly BD, Clarke M, McTigue O, Gervin M, et al. (2005) Incidence and clinical correlates of aggression and violence at presentation in patients with first episode psychosis. Schizophr Res 72: 161-168.

    31. Fresán A, Apiquian R, de la Fuente-Sandoval C, García-Anaya M, Loyzaga C, et al. (2004) Premorbid adjustment and violent behavior in schizophrenic patients Schizophr Res 69: 143-148.

    32. Fresán A, Apiquian R, de la Fuente-Sandoval C, Löyzaga C, García-Anaya M, et al. (2005) Violent behaviour in schizophrenic patients: Relationship with clinical symptoms. Aggressive Behav 31: 511-520.

    33. Fresán A, Apiquian R, Nicolini H, Cervantes JJ (2007a) Temperament and character in violent schizophrenic patients Schizophr Res 94: 74-80.

    34. Fresán A, Apiquian R, García-Anaya M, de la Fuente-Sandoval C, Nicolini H, et al. (2007b) The P50 auditory evoked potential in violent and non-violent patients with schizophrenia. Schizophr Res 97: 128-136.

    35. Fullam RS, Dolan MC (2008) Executive function and in-patient violence in forensic patients with schizophrenia. Br J Psychiatry 193: 247-253.

    36. Guy K, Mohan D, Taylor PJ (2003) Do schizophrenic patients with a history of violence express a preference for screen violence? Int J Forensic Ment Health 2: 165-171.

    37. Hatta K, Takahashi T, Nakamura H, Yamashiro H, Endo H, et al. (1999) The predictive value of benzodiazepine tolerance in persistently aggressive schizophrenia. Neuropsychobiology 39: 196-199.

    38. Herrera JN, Sramek JJ, Costa JF, Roy S, Heh CW, et al. (1988) High potency neuroleptics and violence in schizophrenics. J Nerv Ment Dis 176: 558-561.

    39. Hodgins S, Hiscoke UL, Freese R (2003) The antecedents of aggressive behavior among men with schizophrenia: A prospective investigation of patients in community treatment. Behav Sci Law 21: 523-546.

    40. Hodgins S, Riaz M (2011) Violence and phases of illness: Differential risk and predictors. Eur Psychiatry 26: 518-524.

    41. Hong JP, Lee JS, Chung S, Jung J, Yoo HK, et al. (2008) New functional single nucleotide polymorphism (Ala72Ser) in the COMT gene is associated with aggressive behavior in male schizophrenia. Am J Med Genet Neuropsychiatr Genet 147b: 658-660.

    42. Hoptman MJ, Yates KF, Patalinjug MB, Wack RC, Convit A (1999) Clinical prediction of assaultive behavior among male psychiatric patients at a maximum-security forensic facility. Psychiatr Serv 50: 1461-1466.

    43. Iancu I, Bodner E, Roitman S, Sapir AP, Poreh A, et al. (2010) Impulsivity, aggression and suicide risk among male schizophrenia patients. Psychopathol 43: 223-229.

    44. Jones G, Zammit S, Norton N, Hamshere ML, Jones SJ, et al. (2001) Aggressive behaviour in patients with schizophrenia is associated with catechol-O-methyltransferase genotype. Br J Psychiatry 179: 351-355.

    45. Kim Y-R, Jahng JW, Min SK (2009) Association between the serotonin transporter gene (5-HTTLPR) and anger-related traits in Korean schizophrenic patients Neuropsychobiology 59: 165-171.

    46. Koen L, Kinnear CJ, Corfield VA, Emsley RA, Jordaan E, et al. (2004) Violence in male patients with schizophrenia: Risk markers in a South African population. Aust N Z J Psychiatry 38: 254-259.

    47. Kotler M, Barak P, Cohen H, Averbuch IE, Grinshpoon A, et al. (1999) Homicidal behavior in schizophrenia associated with a genetic polymorphism determining low catechol-O-methyltransferase (COMT) activity. Am J Med Genet Neuropsychiatr Genet 88: 628-633.

    48. Krakowski M, Convit A, Jaeger J, Lin S, Volavka J (1989) Neurological impairment in violent schizophrenic inpatients. Am J Psychiatry 146: 849-853.

    49. Krakowski M, Czobor P, Chou JC-Y (1999) Course of violence in patients with schizophrenia: Relationship to clinical symptoms Schizophr Bull 25: 505-517.

    50. Krakowski M, Czobor P (2004a) Gender differences in violent behaviors: Relationship to clinical symptoms and psychosocial factors. Am J Psychiatry 161: 459-465.

    51. Krakowski M, Czobor P (2004b) Suicide and violence in patients with major psychiatric disorders. J Psychiatr Pract 10: 233-238.

    52. Krakowski M, Czobor P, Citrome L, Bark N, Cooper TB (2006) Atypical antipsychotic agents in the treatment of violent patients with schizophrenia and schizoaffective disorder. Arch Gen Psychiatry 63: 622-629.

    53. Kumari V, Aasen I, Taylor P, ffytche DH, Das M, et al. (2006) Neural dysfunction and violence in schizophrenia: An fMRI investigation. Schizophr Res 84: 144-164.

    54. Kumari V, Das M, Taylor PJ, Barkataki I, Andrew C, et al. (2009a) Neural and behavioural responses to threat in men with a history of serious violence and schizophrenia or antisocial personality disorder. Schizophr Res 110: 47-58.

    55. Kumari V, Barkataki I, Goswami S, Flora S, Das M, et al. (2009b) Dysfunctional, but not functional, impulsivity is associated with a history of seriously violent behaviour and reduced orbitofrontal and hippocampal volumes in schizophrenia. Psychiatry Res Neuroimaging 173: 39-44.

    56. Lachman HM, Nolan KA, Mohr P, Saito T, Volavka J (1998) Association between catechol-O-methyltransferase genotype and violence in schizophrenia and schizoaffective disorder. Am J Psychiatry 155: 835-837.

    57. Lafayette JM, Frankle WG, Pollock A, Dyer K, Goff DC (2003) Clinical characteristics, cognitive functioning, and criminal histories of outpatients with schizophrenia Psychiatr Serv 54 1635-1640.

    58. Lincoln TM, Hodgins S (2008) Is lack of insight associated with physically aggressive behavior among people with schizophrenia living in the community. J Nerv Ment Dis 196: 62-66.

    59. Lysaker PH, Wright DE, Clements CA, Plascak-Hallberg CD (2002) Neurocognitive and psychosocial correlates of hostility among persons in a post-acute phase of schizophrenia spectrum disorders. Compr Psychiatry 43: 319-324.

    60. Maguire K, Cheung P, Crowley K, Norman T, Schweitzer I, et al. (1997) Aggressive behaviour and platelet 3H-paroxetine binding in schizophrenia. Schizophr Res 23: 61-67.

    61. Majorek K, Wolfkühler W, Küper C, Saimeh N, Juckel G, et al. (2009) "Theory of mind" and executive functioning in forensic patients with schizophrenia. J Forensic Sci 54: 469-473.

    62. Martínez-Martín N, Fraguas D, García-Portilla MP, Sáiz PA, Bascarán MT, et al. (2011) Self-percieved needs are related to violent behavior among schizophrenia outpatients. J Nerv Ment Dis 199: 666-671.

    63. Milton J, Amin S, Singh SP, Harrison G, Jones P, et al. (2001) Aggressive incidents in first-episode psychosis. Br J Psychiatry 178: 433-440.

    64. Mitchell LJ (2011) Metacognition in forensic patients with schizophrenia and a history of interpersonal violence: An exploratory study [DClinPsy thesis]. Glasgow: University of Glasgow.

    65. Modai I, Gibel A, Rauchverger B, Ritsner M, Klein E, et al. (2000) Paroxetine binding in aggressive schizophrenic patients. Psychiatry Res 94: 77-81.

    66. Monahan J, Appelbaum PS (2000) Reducing violence risk: Diagnostically based clues from the MacArthur Violence Risk Assessment Study. In: Hodgins S, editor. Effective preventionof crime and violence among the mentally ill. Amsterdam: Kluwer Academic Publishers

    67. Munkner R, Haastrup S, Joergensen T, Kramp P (2005) Incipient offending among schizophrenia patients after first contact to the psychiatric hospital system. Eur Psychiatry 20: 321-326.

    68. Nederlof AF, Muris P, Hovens JE (2011) Threat/control-override symptoms and emotional reactions to positive symptoms as correlates of aggressive behavior in psychotic patients. J Nerv Ment Dis 199: 342-347.

    69. Nolan KA, Volavka J, Mohr P, Czobor P (1999) Psychopathy and violent behavior among patients with schizophrenia or schizoaffective disorder. Psychiatr Serv 50: 787-792.

    70. Nolan KA, Volavka J, Lachman HM, Saito T (2000) An association between a polymorphism of the tryptophan hydroxylase gene and aggression in schizophrenia and schizoaffective disorder. Psychiatr Genet 10: 109-115.

    71. Nolan KA, Volavka J, Czobor P, Sheitman B, Lindenmayer J-P, et al. (2005) Aggression and psychopathology in treatment-resistant inpatients with schizophrenia and schizoaffective disorder. J Psychiatr Res 39: 109-115.

    72. Oldemeyer LN (2009) Demographic and historical factors in violent and nonviolent offenders with psychotic disorders [MSc(Clin Psychol) thesis]. Hillsboro, Oregon: Pacific University.

    73. Ran M-S, Chen P-Y, Liao Z-G, Chan CL-W, Chen EY-H, et al. (2010) Criminal behaviour among persons with schizophrenia in rural China. Schizophr Res 122: 213-218.

    74. Räsänen P, Tiihonen J, Isohanni M, Rantakallio P, Lehtonen J, et al. (1998) Schizophrenia, alcohol abuse, violent behavior: A 26-year followup study of an unselected birth cohort. Schizophr Bull 24: 437-441.

    75. Rasmussen K, Levander S, Sletvold H (1995) Aggressive and non-aggressive schizophrenics: Symptom profile and neuropsychological differences. Psychol Crime Law 2: 119-129.

    76. Ritsner M, Modai I, Gibel A, Leschiner S, Silver H, et al. (2003) Decreased platelet peripheral-type benzodiazapine receptors in persistently violent schizophrenic patients J Psychiatr Res 37: 549-556.

    77. Roy S, Herrera JN, Parent M, Costa JF (1987) Violent and nonviolent schizophrenic patients: Clinical and developmental characteristics. Psychol Rep 61: 855-861.

    78. Sarne Y, Mandel J, Goncalves MH, Brook S, Gafni M, et al. (1995) Imipramine binding to blood platelets and aggressive behavior in offenders, schizophrenics and normal volunteers. Biol Psychiatry 31: 120-124.

    79. Schanda H, Földes P, Topitz A, Fliedl R, Knecht G (1992) Premorbid adjustment of schizophrenic criminal offenders. Acta Psychiatr Scand 86: 121-126.

    80. Schug RA, Yang Y, Raine A, Han C, Liu J (2010) Structural and psychosocial correlates of birth order anomalies in schizophrenia and homicide. J Nerv Ment Dis 198: 870-875.

    81. Serper MR, Beech DR, Harvey PD, Dill C (2008) Neuropsychological and symptom predictors of aggression on the psychiatric inpatient service. J Exp Clin Neuropsychol 30: 700-709.

    82. Silver H, Goodman C, Knoll G, Isakov V, Modai I (2005) Schizophrenia patients with a history of severe violence differ from nonviolent schizophrenia patients in perception of emotions but not cognitive function. J Clin Psychiatry 66: 300-308.

    83. Song H, Min SK (2009) Aggressive behavior model in schizophrenic patients. Psychiatry Res 167: 58-65.

    84. Soyka M, Graz C, Bottlender R, Dirschedl P, Schoech H (2007) Clinical correlates of later violence and criminal offences in schizophrenia. Schizophr Res 94: 89-98.

    85. Spidel A, Lecomte T, Greaves C, Sahlstrom K, Yuille JC (2010) Early psychosis and aggression: Predictors and prevalence of violent behaviour amongst individuals with early onset psychosis. Int J Law Psychiatry 33: 171-176.

    86. Steinert T, Hermer K, Faust V (1996) Comparison of aggressive and non-aggressive schizophrenic inpatients matched for age and sex. Eur J Psychiatr 10: 100-107.

    87. Steinert T, Wiebe C, Gebhardt RP (1999) Aggressive behavior against self and others among first-admission patients with schizophrenia. Psychiatr Serv 50: 85-90.

    88. Stompe T, Ortwein-Swoboda G, Schanda H (2004) Schizophrenia, delusional symptoms and violence: The threat/control-override concept reexamined. Schizophr Bull 30: 31-44.

    89. Stompe T, Strnad A, Ritter K, Fischer-Danzinger D, Letmaier M, et al. (2006) Family and social influences on offending in men with schizophrenia. Aust N Z J Psychiatry 40: 554-560.

    90. Swanson JW, Swartz MS, Borum R, Hiday VA, Wagner R, et al. (2000) Involuntary out-patient commitment and reduction of violent behaviour in persons with severe mental illness. Br J Psychiatry 176: 324-331.

    91. Swanson JW, Swartz MS, Essock SM, Osher FC, Wagner HR, et al. (2002) The social-environmental context of violent behavior in persons treated for severe mental illness. Am J Public Health 92: 1523-1531.

    92. Swanson J, Swartz M, Elbogen E (2004) Effectiveness of atypical antipsychotic medications in reducing violent behavior among persons with schizophrenia in community-based treatment. Schizophr Bull 30: 3-20.

    93. Swanson JW, Van Dorn RA, Monahan J, Swartz MS (2006) Violence and leveraged community treatment for persons with mental disorders. Am J Psychiatry 163: 1404-1411.

    94. Swanson JW, Swartz MS, Van Dorn RA, Elbogen EB, Wagner HR, et al. (2006) A national study of violent behavior in persons with schizophrenia. Arch Gen Psychiatry 63: 490-499.

    95. Swanson JW, Van Dorn RA, Swartz MS, Smith A, Elbogen EB, et al. (2008) Alternative pathways to violence in persons with schizophrenia: The role of childhood antisocial behavior problems. Law Hum Behav 32: 228-240.

    96. Teixeria EH, Dalgalarrondo P (2009) Violent crime and dimesions of delusion: A comparative study of criminal and noncriminal delusional patients. J Am Acad Psychiatry Law 37: 225-231.

    97. Thomas S, Leese M, Walsh E, McCrone P, Moran P, et al. (2005) A comparison of statistical models in predicting violence in psychotic illness. Compr Psychiatry 46: 296-303.

    98. Tripathi RK, Jahan M (2010) 16 PF profile of schizophrenics with and without criminal record. Psychol Stud 55: 351-357.

    99. Tuninger E, Levander S, Bernce R, Johansson G (2001) Criminality and aggression among psychotic in-patients: Frequency and clinical correlates. Acta Psychiatr Scand 103: 294-300.

    100. Turkoglu A, Tokdemir M, Atmaca M, Namli M, Ustundag B (2009) Serum cholesterol, triglyceride, and ghrelin levels in criminal and non-criminal schizophrenia patients. Bull Clin Psychopharmacol 19: 353-358.

    101. Valevski A, Averbuch IE, Radwan M, Gur S, Spivak B, et al. (1999) Homicide by schizophrenic patients in Israel. Eur Psychiatry 14: 89-92.

    102. Verma S, Poon LY, Subramaniam M, Chong S-A (2005) Aggression in Asian patients with first-episode psychosis. Int J Soc Psychiatry 51: 365-371.

    103. Vevera J, Hubbard A, Veselý A, Papežová H (2005) Violent behaviour in schizophrenia Br J Psychiatry 187: 426-430.

    104. Volavka J, Laska E, Baker S, Meisner M, Czobor P, et al. (1997) History of violent behaviour and schizophrenia in different cultures: Analyses based on the WHO study on Determinants of Outcome of Severe Mental Disorders. Br J Psychiatry 171: 9-14.

    105. Wootton L, Buchanan A, Leese M, Tyrer P, Burns T, et al. (2008) Violence in psychosis: Estimating the predictive validity of readily accessible clinical information in a community sample. Schizophr Res 101: 176-184.

    106. Yang Y, Raine A, Han C-B, Schug RA, Toga AW, et al. (2010) Reduced hippocampal and parahippocampal volumes in murderers with schizophrenia. Psychiatry Res Neuroimaging 182: 9-13.

    107. Yen C-F, Yeh M-L, Chen C-S, Chung H-H (2002) Predictive value of insight for suicide, violence, hospitalization and social adjustment for outpatients with schizophrenia: A prospective study Compr Psychiatry 43: 443-447.

    108. Yesavage JA (1983) Inpatient violence and the schizophrenic patient: A study of the Brief Psychiatric Rating Scale scores and inpatient behavior. Acta Psychiatr Scand 67: 353-357.

    109. Yesavage JA (1984) Correlates of dangerous behavior by schizophrenics in hospital. J Psychiatr Res 18: 225-231.

    110. Zoghes D, Liakos A, Potamianos G (1988) An investigation into the relationship between hostility and criminality in schizophrenia. Int J Soc Psychiatry 34: 207-211. [↑](#endnote-ref-26)
