# Supplementary material for: Risk Factors for Violence in Psychosis: Systematic Review and Meta-Regression Analysis of 110 Studies
Source: PLoS One. 2013 Feb 13;8(2):e55942. doi: 10.1371/journal.pone.0055942 (PMC3572179; doi:10.1371/journal.pone.0055942)
Supplement: Table S3 — Beta coefficients, standard errors and probability values for the 34 risk and protective factors measured on a continuous scale in which Egger’s test of publication bias was significant. (DOCX) [file pone.0055942.s005.docx]

**Table S3.** Beta coefficients, standard errors and probability values for the 34 risk and protective factors measured on a continuous scale in which Egger’s test of publication bias was significant.

| **Risk Domain** | | **Risk Factor** | **Egger’s Test of Publication Bias** | | |
| --- | --- | --- | --- | --- | --- |
|  |  |  | ***β*** | ***se*** | ***p*** |
| **Demographic** | |  |  |  |  |
|  | Shorter duration of education (years) | | 0.5 | 0.1 | 0.001 |
|  | Younger age at study enrolment (years) | | 0.04 | 0.03 | <0.001 |
| **Premorbid** | |  |  |  |  |
|  | Higher premorbid adjustment in early adolescence scores | | -0.2 | 0.007 | 0.01 |
| **Criminal History** | |  |  |  |  |
|  | Higher scores on the Aggression Against Others subscale | | -1.5 | 0.1 | 0.03 |
|  | Higher aggression scores | | -1.3 | 0.03 | <0.001 |
|  | Higher psychopathy factor 1 scores | | -1.9 | 0.1 | 0.02 |
|  | Higher verbal aggression scores | | -1.7 | 0.1 | <0.001 |
|  | Higher psychopathy total scores | | -0.4 | 0.1 | 0.001 |
|  | Greater number of arrests (any offence) | | -0.1 | 0.05 | 0.01 |
|  | Higher scores on the Aggression Against Objects subscale | | -1.0 | 0.08 | 0.003 |
|  | Higher poor hostile and/or aggressive impulse control scores | | -1.6 | 0.05 | 0.01 |
|  | Higher hostility scores | | -0.6 | 0.2 | <0.001 |
|  | Younger age at first criminal offence | | 0.2 | 0.01 | 0.01 |
| **Psychopathology** | |  |  |  |  |
|  | Higher poor impulse control scores | | -0.6 | 0.1 | <0.001 |
|  | Higher preoccupation scores | | -1.2 | 0.01 | 0.005 |
|  | Higher scores on the Lack of Insight Mental Disorder subscale | | -0.8 | 0.08 | 0.03 |
|  | Higher lack of insight/judgement scores | | -0.4 | 0.06 | <0.001 |
|  | Higher cognitive functioning scores | | -0.5 | 0.07 | 0.001 |
|  | Higher total PANSS scores | | -0.3 | 0.2 | 0.003 |
|  | Higher guilt scores | | -0.4 | 0.07 | 0.006 |
|  | Higher somatic concerns scores | | -0.2 | 0.03 | <0.001 |
|  | Higher uncooperativeness scores | | -0.3 | 0.1 | 0.002 |
|  | Higher confusion/disorientation scores | | -0.1 | 0.04 | 0.001 |
|  | Higher total BPRS scores | | -1.1 | 0.1 | 0.001 |
|  | Younger age at psychosis onset (years) | | 0.2 | 0.1 | 0.03 |
| **Positive Symptoms** | |  |  |  |  |
|  | Higher excitement scores | | -0.4 | 0.1 | <0.001 |
|  | Higher positive symptoms scores | | -0.7 | 0.1 | <0.001 |
|  | Higher grandiosity scores | | -0.2 | 0.04 | 0.001 |
|  | Higher suspiciousness/persecution scores | | -0.3 | 0.1 | 0.006 |
|  | Higher paranoia scores | | 4.7 | 0.2 | 0.03 |
| **Negative Symptoms** | |  |  |  |  |
|  | Lower total Quality of Life scores | | 0.3 | 0.03 | 0.03 |
|  | Higher negative symptoms scores | | -0.1 | 0.07 | 0.02 |
| **Treatment-Related** | |  |  |  |  |
|  | Shorter duration of current outpatient treatment (months) | | 0.3 | 0.002 | 0.002 |
|  | Younger age at first psychiatric inpatient admission | | 0.2 | 0.04 | 0.007 |
